# Prenatal Metformin Exposure in a Maternal High Fat Diet Mouse Model Alters the Transcriptome and Modifies the Metabolic Responses of the Offspring

**DOI:** 10.1371/journal.pone.0115778

**Published:** 2014-12-26

**Authors:** Henriikka Salomäki, Merja Heinäniemi, Laura H. Vähätalo, Liisa Ailanen, Kim Eerola, Suvi T. Ruohonen, Ullamari Pesonen, Markku Koulu

**Affiliations:** 1 Department of Pharmacology, Drug Development and Therapeutics, Institute of Biomedicine, University of Turku, FI-20014 Turku, Finland; 2 Drug Research Doctoral Programme (DRDP), University of Turku, Turku, Finland; 3 School of Medicine, Institute of Biomedicine, University of Eastern Finland, FI-70211 Kuopio, Finland; 4 Turku Center for Disease Modeling (TCDM), University of Turku, FI-20014 Turku, Finland; Institut d'Investigacions Biomèdiques August Pi i Sunyer, Spain

## Abstract

**Aims:**

Despite the wide use of metformin in metabolically challenged pregnancies, the long-term effects on the metabolism of the offspring are not known. We studied the long-term effects of prenatal metformin exposure during metabolically challenged pregnancy in mice.

**Materials and Methods:**

Female mice were on a high fat diet (HFD) prior to and during the gestation. Metformin was administered during gestation from E0.5 to E17.5. Male and female offspring were weaned to a regular diet (RD) and subjected to HFD at adulthood (10-11 weeks). Body weight and several metabolic parameters (e.g. body composition and glucose tolerance) were measured during the study. Microarray and subsequent pathway analyses on the liver and subcutaneous adipose tissue of the male offspring were performed at postnatal day 4 in a separate experiment.

**Results:**

Prenatal metformin exposure changed the offspring's response to HFD. Metformin exposed offspring gained less body weight and adipose tissue during the HFD phase. Additionally, prenatal metformin exposure prevented HFD-induced impairment in glucose tolerance. Microarray and annotation analyses revealed metformin-induced changes in several metabolic pathways from which electron transport chain (ETC) was prominently affected both in the neonatal liver and adipose tissue.

**Conclusion:**

This study shows the beneficial effects of prenatal metformin exposure on the offspring's glucose tolerance and fat mass accumulation during HFD. The transcriptome data obtained at neonatal age indicates major effects on the genes involved in mitochondrial ATP production and adipocyte differentiation suggesting the mechanistic routes to improved metabolic phenotype at adulthood.

## Introduction

Metformin, a biguanide class anti-diabetic agent, is the most commonly prescribed oral drug for type 2 diabetes. Its main pharmacological effect in type 2 diabetes is mediated by decreased hepatic gluconeogenesis [1], [2]. Additionally, metformin has been shown to improve insulin sensitivity, lipid profile, endothelial function, body weight control and reduce inflammation [2]–[4]. There is evidence that metformin acts *via* AMP-activated protein kinase (AMPK) at cellular level [5], [6]. Nevertheless, recent data has described that the glucose-lowering effect of metformin may not recruit AMPK in all cases [7] which demonstrate that the knowledge of metformin's molecular and cellular mechanisms are still incomplete.

The prevalence of obesity and obesity-associated diseases has increased not only in adults, but also in children and adolescents [8]. In 2010, an estimated 42 million children under the age of five were overweight worldwide [9]. The trend in obesity development has been associated with trans-generational effects (i.e. fetal programming) as diabetic environment *in utero* can predispose the offspring to impaired metabolism starting already at childhood [10]–[13]. Thus, metabolically challenged pregnancies clearly benefit from active intervention [14]. Insulin is commonly used to decrease many of the diabetes-associated complications in gestational diabetes mellitus (GDM). However, insulin treatment increases the risk of hypoglycemia and might also result in poor compliance.

Metformin was introduced to the treatment of GDM for the first time already in the 1970's [15]. At present, clinical data exists of the use of metformin in GDM and in pregnancies complicated by polycystic ovary syndrome (PCOS) [16]–[21]. These results show improvements to routine care. The role of metformin treatment during pregnancy has however remained disputable due to the lack of information of the long-term effects of the maternal drug treatment on the offspring.

Rodent models have been utilised extensively to investigate fetal programming induced by maternal malnutrition, stress, obesity, obesogenic diet and hyperglycemia [22], [23]. We have previously studied the long-term effects of prenatal metformin exposure with pregnant mice on a regular diet without any metabolic challenge during the gestation [24]. In this model the metformin exposed offspring had increased adipose tissue accumulation and impaired glucose tolerance during HFD at adulthood. However, since the metabolic status of the pregnant mice was normal, this model might not reflect the clinical use of metformin during pregnancy. Therefore we wanted to test our hypothesis of the programming effect of prenatal metformin exposure in a metabolically challenged model. In the current study, we administered metformin from E0.5 until E17.5 to female mice which were challenged with HFD prior to and during the gestation to mimic the metabolic situation in which metformin treatment is used in humans. Importantly, the offspring were followed until adulthood to characterise their future metabolic phenotype. In addition, we performed a microarray analysis on the liver and adipose tissue of the male offspring at postnatal day 4 to reveal any early changes in the gene expression pattern.

## Materials and Methods

### Ethics statement

Animal work was planned and performed according to the Finnish Act on the Use of Animals for Experimental Purposes. The study scheme was approved by the Finnish Animal Experiment Board (Permit ESAVI-2010-06188). During the studies, the mice were monitored for any signs of morbidity and all efforts were made to minimise suffering.

### Mice and diets

C57/BL6NHsd mice (Harlan Laboratories, NL) were housed on a 12 h:12 h dark:light cycle and fed either a regular diet (i.e. RD; 69 kcal% carbohydrates, 22 kcal% protein and 9 kcal% fat resulting in digestible energy of 2.97 kcal/g and metabolisable energy of 2.71 kcal/g; CRM(E), SDS, UK) or high fat diet (i.e. HFD; 20 kcal% carbohydrates, 20 kcal% protein and 60 kcal% of fat resulting in total energy of 5.24 kcal/g; D12492, Research Diets, NJ, USA) *ad libitum*.

### Study design

**Figure 1:**
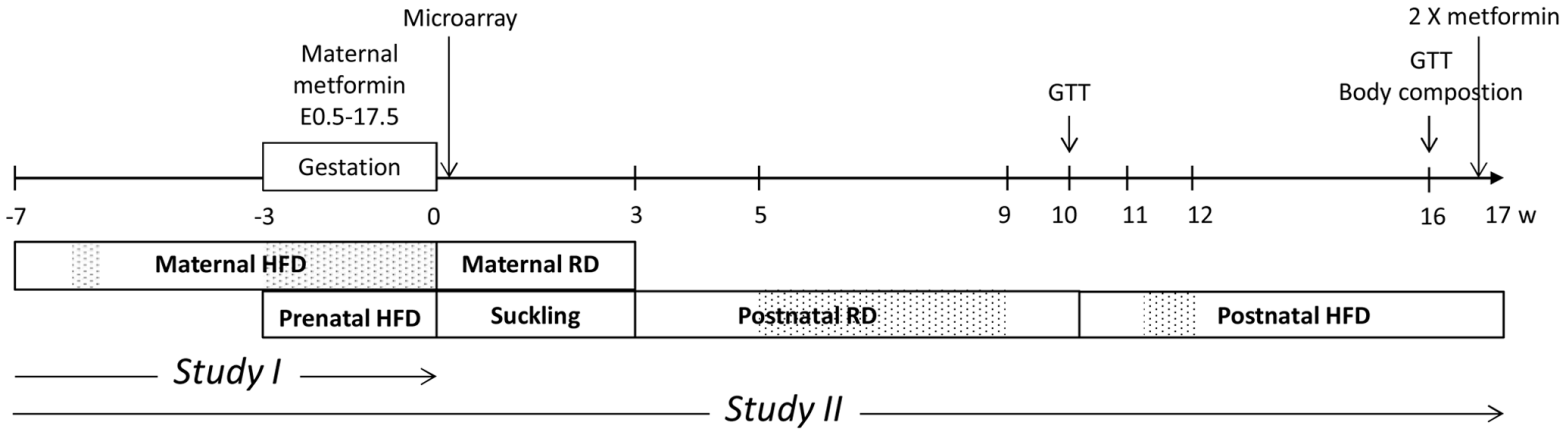
Study design. Female mice were fed with HFD prior to (weeks −7– −3) and during the gestation. Metformin was administered from E0.5 to 17.5. Week 0 denotes the birth of the offspring. The data were obtained from two separate set of mice, study I and II as indicated below the timeline. Study I: gestational glucose measurement, microarray samples and a surrogate group until E18.5; Study II: the long-term follow of the offspring and a surrogate group until E18.5. Only male offspring were used for the microarray analyses. At the end of the study, the offspring in the long-term study II were subjected to an acute metformin administration (2×300 mg/kg). Dotted areas  =  periods of food intake measurement. GTT =  glucose tolerance test.

Female mice (age 7–8 weeks) were placed on a HFD for one month prior to mating and they were maintained on the HFD during the gestation. Metformin (Sigma-Aldrich, St. Louis, MO, USA) was administered p.o. (300 mg/kg) to the dams from E0.5 to E17.5 as described previously [24]. The dose of metformin was selected according to previous studies [24]–[29]. Food intake was measured daily during the dosing. The diet was gradually changed to RD by mixing RD and HFD diet from E18.5 and changing the diet solely to RD after the parturition for the dams to acclimatise. Control groups fed with RD were included for the pre-gestational time period and for the gestation. The long-term follow-up was performed with adjusted litter sizes 6–10 (pups from six dams in both groups). At the age of three weeks, offspring were weaned to RD. From the age of 10–11 weeks until 17 weeks the offspring continued on HFD. Body weight was recorded once a week. Food intake was measured during the experiment in cages inhabited by 2–5 mice. At the study endpoint, the offspring of metformin and vehicle treated dams were randomised to receive a dose of metformin (300 mg/kg p.o.) or vehicle 24 hours and one hour prior to tissue collection to study whether the long-term prenatal metformin exposure affects the response to a later acute exposure. Terminal anesthesia was induced with a mixture of medetomidine and ketamine in saline as described previously [24]. Surrogate groups until E18.5 (birth weight) and postnatal day 4 were included in the study. At postnatal day 4, subcutaneous adipose tissue (SAT) and liver was collected from the male offspring for the microarray analyses. Study design and timeline are shown in Fig. 1.

### Blood glucose measurements and glucose tolerance test (GTT)

Maternal blood glucose was measured (Precision Xceed, Abbot Diabetes Care Ltd, Witney, Oxon, UK) on the second week of gestation (E11.5–12.5) after a 3–4 -hour fast and administration of metformin (300 mg/kg p.o.) or vehicle. Prior to tissue collection, blood glucose of the offspring was measured one hour from the last acute metformin dose.

GTT for the offspring were performed at the age of 10 weeks (RD phase) and 15–16 weeks (HFD phase). Following a 4-hour fast, the basal level of blood glucose was measured. Glucose (1 g/kg) was injected i.p. and the blood glucose concentration was measured from a blood sample obtained from the tail vein 20, 40, 60 and 90 minutes after the glucose injection.

### Body composition

Body composition was measured by quantitative nuclear magnetic resonance (NMR) scanning (EchoMRI-700, Echo Medical Systems) at the age of 16 weeks during the HFD as described previously [24].

### Tissue collection and serum lipid and adipokine quantifications

Serum was obtained *via* cardiac puncture under terminal anesthesia. Subsequent to decapitation, following tissues were collected and weighed: liver, inguinal (iWAT), gonadal/epididymal (gWAT and eWAT, respectively), mesenteric (mWAT) and retroperitoneal white adipose tissue (rWAT). Serum triglycerides, non-esterified fatty acids (NEFA) and total cholesterol were quantified as described previously [24]. Serum leptin, resistin and insulin were measured with Mouse Adipokine Magnetic Bead Panel (EMD Millipore Corporation, MA, USA) in single replicate or duplicates.

### Adipocyte size analysis

Adipocyte size was determined from the gWAT of the female offspring at the study endpoint. 4 µm sections were prepared form paraffin-embedded gWAT and stained with hematoxylin and eosin. The slides were scanned with Pannoramic 250 Whole Slide Digital Scanner (PerkinElmer, MA, USA) and 20× images were taken with Pannoramic Viewer (www.3dhistech.com/pannoramic_viewer). The areas of the adipocytes were quantified by a semi-automated method described in Parlee et al. [30] with minor modifications. RGB-images were split in ImageJ (http://imagej.nih.gov/ij/) to red, green and blue colour channels from which the green channel was selected for further analysis. Background and noise substraction were performed accordingly [30], and the threshold was adjusted to 250. Six out of 25 samples were analysed with two grades higher threshold (252) due to a lower level of staining signal. After the binary transformation of the image the colours were inverted and masks of the adipocytes were created with Analyze Particles -command with the following criteria: circularity of 0.35–1, size from 350 µm^2^ to infinity, exclude edges [30]. Five representative 20× images or at least 100 cells were counted from each mouse (sample size 137±18 and 155±12 in Ctr and Met, respectively). The frequency distribution was performed with frequency function (Microsoft Excel) in the scale of 0–15000 µm^2^ on every 500 µm^2^. The volumes of the adipocytes were calculated by assuming a circular and spherical form. The volume of the gWAT depot was calculated based on the adipose tissue density (0.9 g/ml).

### Illumina microarray analyses

Samples were obtained from the liver and SAT of 4-day old male offspring in the surrogate group. Total RNA was isolated according to the manufacturer's instructions (Qiagen RNAeasy Mini Kit and miRNeasy Micro Kit) with an additional DNase I digestion step. For the microarray, 400 ng of total RNA was amplified with Illumina TotalPrep-96 RNA Amplification Kit (Applied Biosystems). cRNA was hybridised to the Illumina Sentrix Mouse WG-6 v2 Expression Bead Chip -microarray chip. Hybridisation was detected with Cyanine3-strepavidin. The microarray data was analysed using the R software package (http://www.r-project.org/) and the Bioconductor lumi and limma packages (www.bioconductor.org) in combination with our previous microarray dataset [24] to increase the power of the analysis relevant for the liver data. Following variance stabilization transformation and robust splines normalization (available as lumiT and lumiN functions in the lumi package), the quality of samples was assessed using boxplots. Sample grouping according to experimental groups was ascertained using multidimensional scaling. One outlier sample with considerably lower signal value distribution from the high fat diet metformin SAT group was discarded. From the remaining data matrix, probes with a detection P-value <0.05 in at least one sample group were included to the analysis. For each comparison, differentially expressed genes were identified using moderated t-statistics with a false discovery rate <0.05 (Benjamini-Hochberg method, available in the limma package). Using the list of differentially expressed genes, the enrichment of functional terms from KEGG and REACTOME databases and genes characteristic of the browning phenotype of adipocytes (*Prdm16, Ucp1, CIDEA, Cox8b, GLUT4, Ppargca1, Elovl3, Fabp4, AdipoQ)* [31] was analysed using the hypergeometric test available in the Graphite Web tool (http://graphiteweb.bio.unipd.it/).

### Quantitative Real-Time PCR (qPCR)

To validate the microarray data, 500 ng of the RNA was transcribed to cDNA using SuperScript VILO (Life Technologies) cDNA synthesis kit. qPCR was performed using the SYBR green (Kapa Biosystems, MA, USA) method in a ABI 7300 Real Time PCR system. The final concentration of forward and reverse primers in the reaction was 0.2 µM. The data was analysed according to the 2^−ΔΔCt^ method using *Rps29* as a reference gene (relative expression to *Rps29*). Primer sequences are shown in S1 Table.

### Statistical analyses

Body weight development, food intake and glucose tolerance were analysed with 2-way RM-ANOVA with Tukey's and Sidak's *post-hoc* tests. Regular 2-way ANOVA with Tukey's and Sidak's *post-hoc* tests was used for adipocyte size distribution and endpoint analyses after the acute metformin dosage. In order to compare two groups with continuous variables, Student's t-test or Mann-Whitney test was used. Statistical analyses were performed with GraphPad Prism 6.0. The data are expressed as mean ±SEM. Results were considered statistically significant if P<0.05.

## Results

### The effect of HFD and metformin treatment on the dams and weight of the fetuses (E18.5)

**Figure 2:**
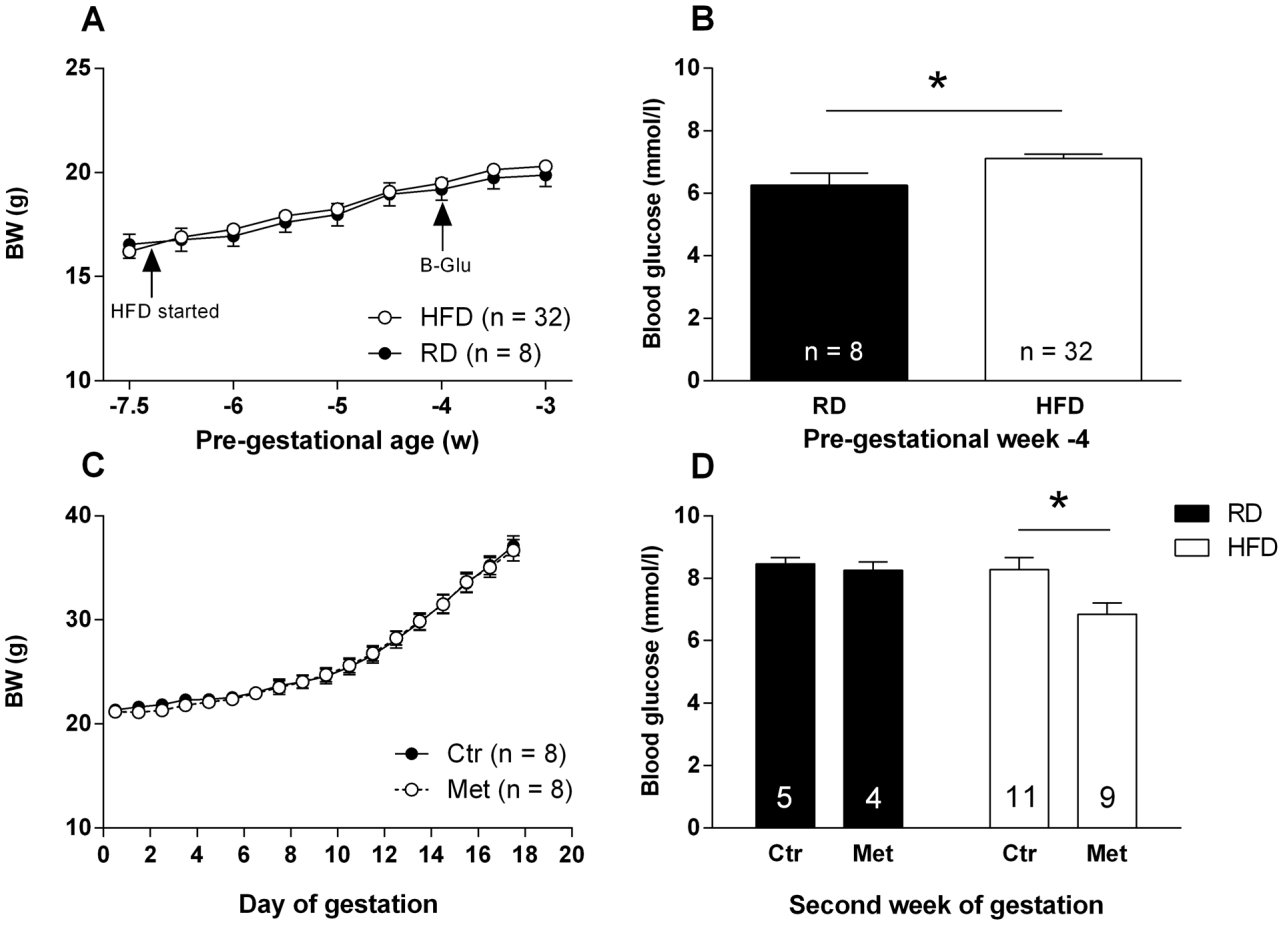
The effect of HFD and metformin treatment on the metabolism of the dams. Body weight during pre-gestational HFD period (A). Fasting blood glucose after three weeks of HFD consumption (B). Body weight development during E0.5–17.5 (C). Blood glucose on the second week (E11.5–12.5) of the gestation (D). * = P<0.05 by Student's t-test.

Body weight development of the female mice was similar between RD and HFD feeding groups during the pre-gestational period (Fig. 2A), although the caloric intake tended to be higher in the HFD group based on a 4-day follow-up (P = 0.061; data not shown). Moreover, HFD elevated the fasting blood glucose level compared to the RD group (Fig. 2B).

**Figure 3:**
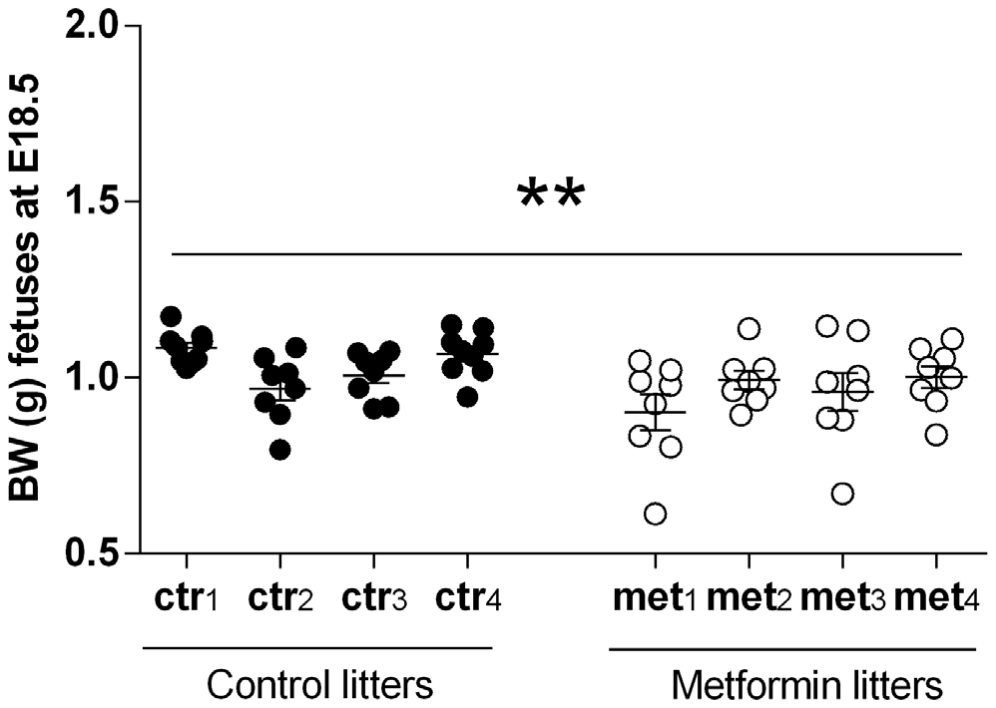
Weight of the fetuses (E18.5). Body weight of litters at E18.5; ctr1-4 denotes prenatal control litters and met1-4 prenatal metformin litters, Litter size 8–9. ** = P<0.01 by Mann-Whitney test when individual fetal weights are tested (pooled results from two separate studies).

During the gestation, metformin and vehicle treated HFD dams gained weight similarly (Fig. 2C) and their food intake was equal (data not shown). After 3–4 hours of oral metformin administration, the fasting blood glucose was decreased compared to the vehicle treated dams on HFD examined on the second week of gestation (E11.5–12.5; Fig. 2D). In the RD group no blood glucose lowering effect of metformin was observed. Furthermore, the fetuses exposed to metformin were significantly (P<0.01) lighter at E18.5 compared to those of vehicle treated dams (Fig. 3).

### The metabolic effects of prenatal metformin exposure on the offspring

**Figure 4:**
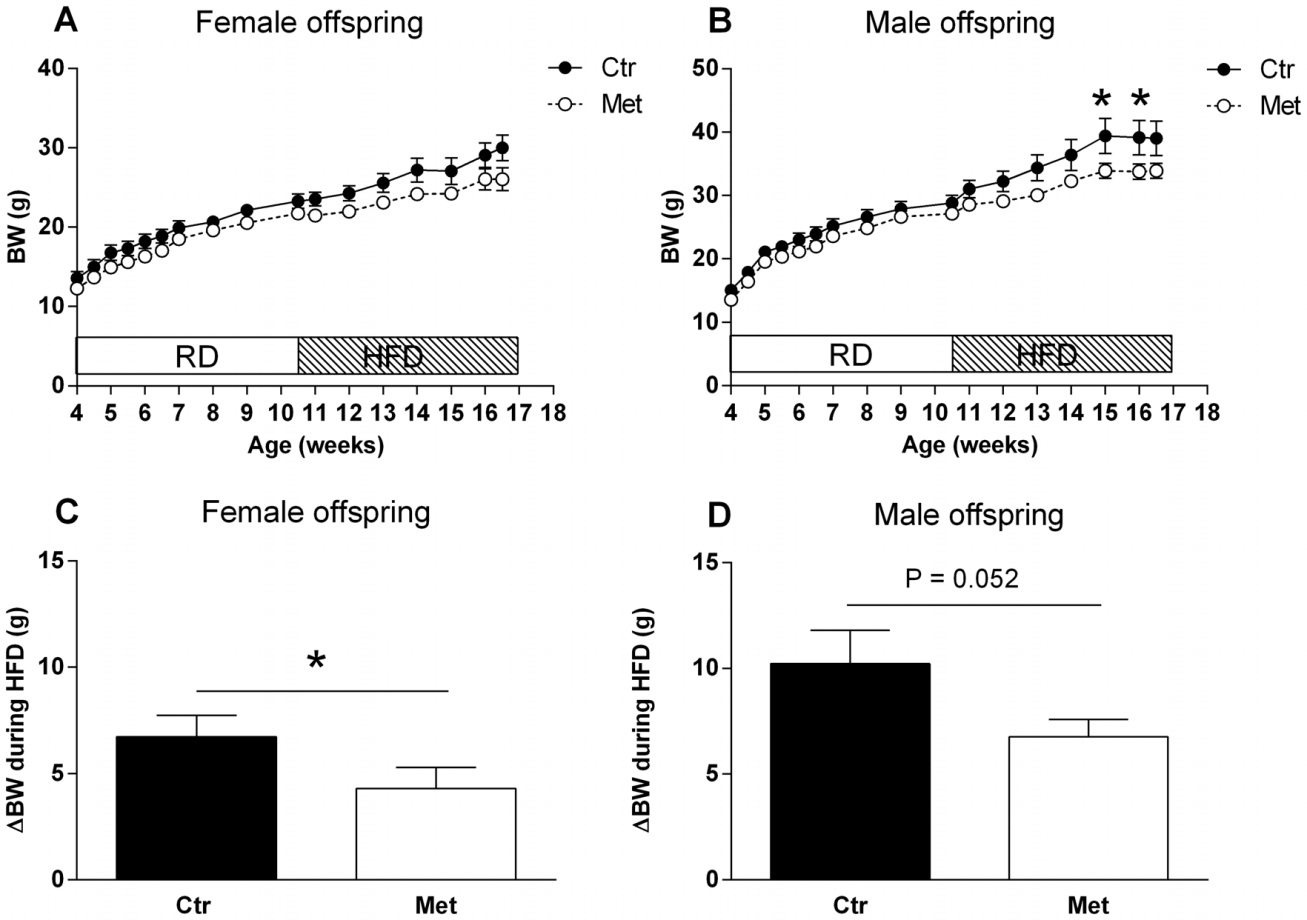
The effect of prenatal metformin exposure on the body weight of the offspring during RD and HFD phases. Female (A) and male offspring (B) during RD and HFD. Black circles (•)  =  Control offspring, open circles (○)  =  Metformin offspring. * = P<0.05 by 2-way RM-ANOVA and Sidak's multiple comparisons test. The body weight change during the HFD in the female (C) and male (D) offspring. n = 13–16, * = P<0.05 by Mann-Whitney test.

Body weights of the control and metformin offspring did not differ during the RD phase in either gender (Fig. 4A–B). Additionally, there was no difference in the food intake during the RD phase (5–9 weeks, data not shown).

During the HFD, metformin exposed offspring gained less body weight and adipose tissue. The net body weight change during the HFD phase in the female control and metformin offspring was 6.7 g±1.0 g and 4.3 g±1.0 g, respectively (P<0.05; Fig. 4C). In the male offspring the net body weight change in the control offspring was 10.2 g±1.6 g and 6.8 g±0.8 g in the metformin offspring (P = 0.052; Fig. 4D). Female metformin offspring had also significantly reduced fat% (P<0.01) and increased lean mass% (P<0.05) after six weeks of HFD (Table 1) and a decreased weight in all WAT depots weighed one week subsequent to the NMR analysis (Table 2). Metformin exposed male offspring had tendency (P = 0.09) to lower total adipose tissue mass in grams (NMR scan, Table 1) and to mWAT weight (P = 0.08; Table 2). On the other hand, liver weight was decreased in the metformin exposed male offspring but not in the female offspring (Table 2). There were no significant differences in the food intake during seven consecutive days recorded one week after the start of the HFD. An average cumulative food intake in the control and metformin offspring during the period was 105±7 kcal vs. 97±3 kcal (P = 0.354) in the males and 93±8 kcal vs. 112±11 kcal (P = 0.258) in the females.

**Table 1:** Body composition analysis.

	Female offspring			Male offspring		
	Ctr	Met	P	Ctr	Met	P
Body composition	n = 13	n = 16		n = 13	n = 16	
Fat mass (g)	8.2±1.1	5.2±0.8	<0.05	11.4±1.8	8.0±1.0	*NS*
Fat mass (%)	26.8±2.3	18.6±1.8	<0.05	26.9±2.8	22.4±2.1	*NS*
Lean mass (g)	18.6±0.5	18.3±0.5	*NS*	24.5±0.9	23.0±0.4	*NS*
Lean mass (%)	65.0±2.1	71.5±1.6	<0.05	64.6±2.7	68.5±1.9	*NS*
Total water (%)	52.8±2.2	58.6±1.7	<0.05	52.4±2.7	55.0±2.0	*NS*

Fat mass (g, %), lean mass (g, %) and total water (%) in the female and male offspring by NMR scanning after six weeks of HFD consumption. P <0.05 and <0.01 by Student's t-test. *NS* =  not significant.

**Table 2:** Absolute (g) and relative (% of BW) tissue weights of the offspring.

	Female offspring			Male offspring		
	Ctr	Met	P	Ctr	Met	P
**Tissue weight (g)**	**n = 13**	**n = 16**		**n = 13**	**n = 16**	
iWAT	0.632±0.088	0.338±0.068	<0.05	0.691±0.131	0.427±0.064	*NS*
gWAT/eWAT	0.821±0.122	0.409±0.082	<0.01	1.181±0.184	0.873± 0.126	*NS*
rWAT	0.431±0.069	0.205±0.046	<0.01	0.415±0.064	0.422±0.088	*NS*
mWAT	0.583±0.084	0.331±0.066	<0.05	0.967±0.205	0.603±0.073	*NS*
Liver	1.165±0.101	1.078±0.06	*NS*	1.616±0.134	1.318±0.059	<0.05
**Tissue weights relative to body weight (%)**						
iWAt	2.0 ±0.2	1.2±0.2	<0.05	1.6±0.2	1.3±0.1	*NS*
gWAT/eWAT	2.6±0.3	1.5±0.2	<0.01	2.8±0.3	2.4±0.3	*NS*
rWAT	1.3±0.2	0.7±0.1	<0.01	1.0±0.1	1.2±0.2	*NS*
mWAT	1.9±0.2	1.1±0.2	<0.05	2.2±0.4	1.7±0.2	*NS*
Liver	3.9±0.2	4.2±0.09	*NS*	4.1±0.1	3.9±0.1	*NS*

The weights (g) of iWAT, g/eWAT, rWAT and mWAT and liver after seven weeks of HFD consumption (upper rows of the table). Tissue weights relative to the body weight (%; lower rows of the table). P-values by Student's t-test or Mann-Whitney test. *NS* =  not significant.

### The adipocyte size distribution in gWAT of the female offspring

**Figure 5:**
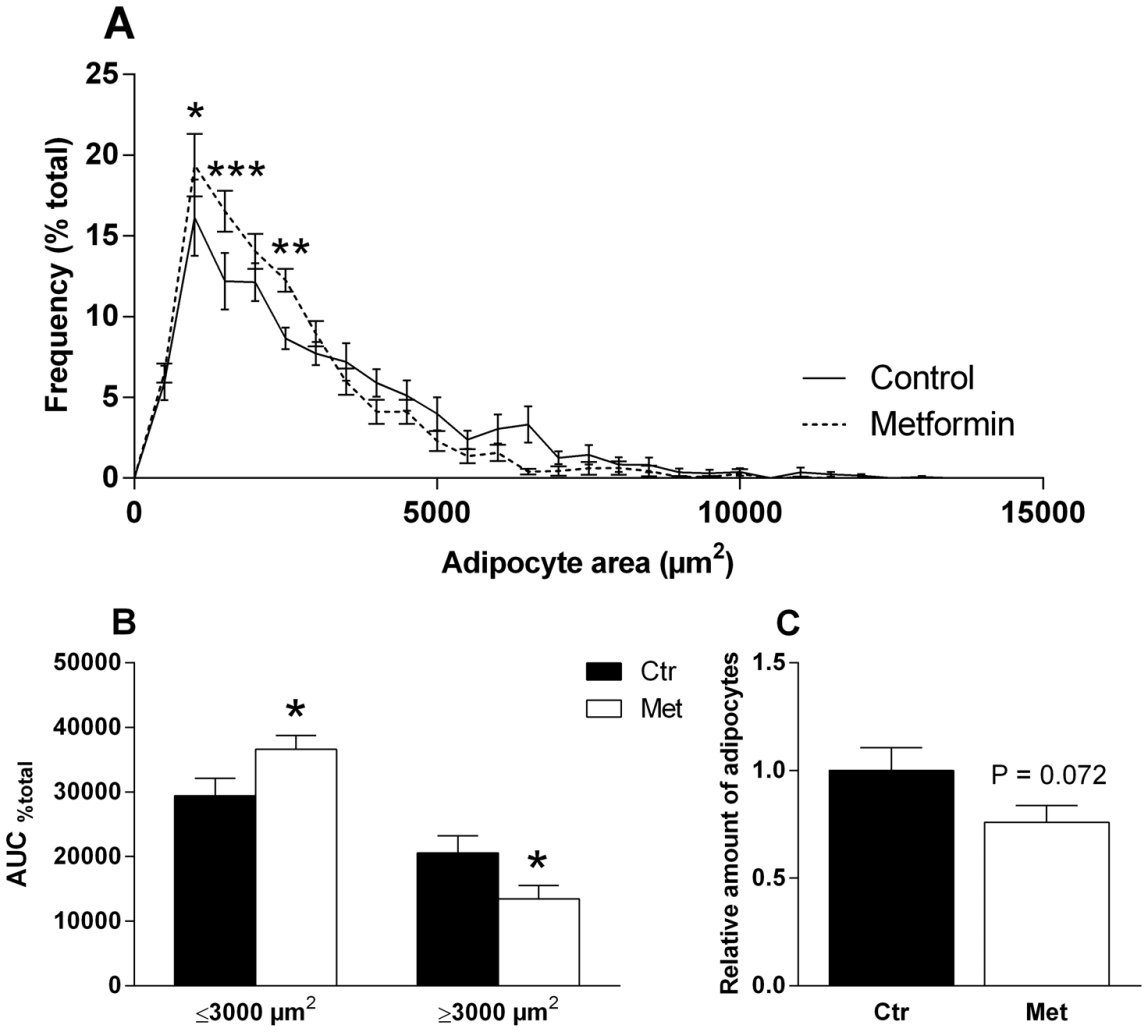
Adipocyte size distribution in gWAT at the end of the postnatal HFD phase. The percentage of the adipocytes on every 500 µm^2^. * = P<0.05, ** = P<0.01 and *** = P<0.001 by 2-way ANOVA and Sidak's multiple comparisons test (A). AUC of the adipocyte size distribution curve ≤3000 µm^2^ and ≥ 3000 µm^2^ (B). Relative amount of adipocytes (C). n = 11–14. * = P<0.05 by Student's t-test.

The adipocyte size distribution was different in the female control and metformin offspring measured at the end of the HFD phase (Fig. 5A). Metformin offspring had a higher percentage of adipocytes ≤3000 µm^2^ and smaller percentage of adipocytes ≥3000 µm^2^ (Fig. 5B). Furthermore, a calculated estimation of the number of adipocytes in the gWAT depots demonstrated that there was a strong tendency (P = 0.072) to hypoplasia in the metformin offspring (Fig. 5C).

### Prenatal metformin exposure in glucose tolerance during RD and HFD

**Figure 6:**
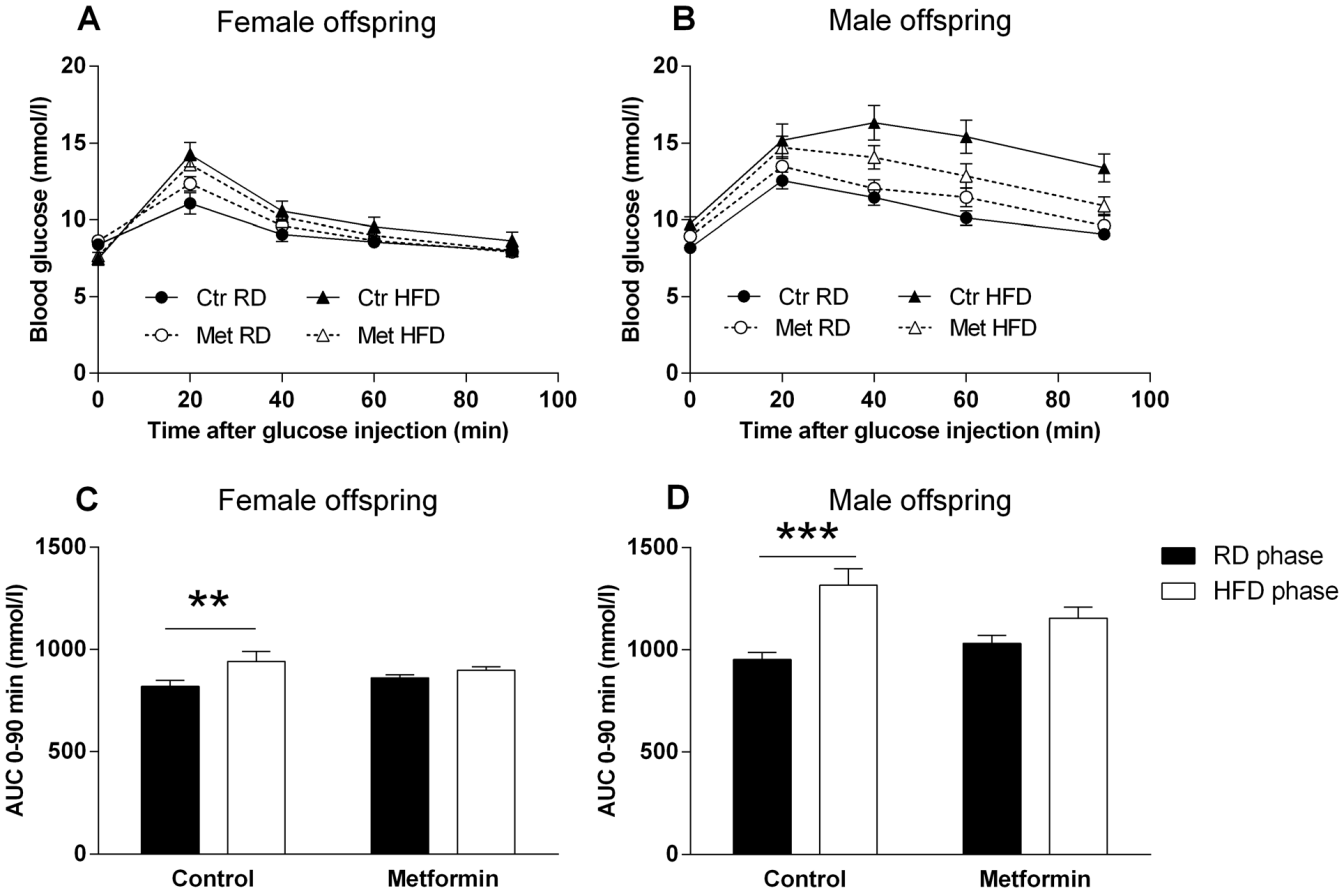
Glucose tolerance of the offspring during RD and HFD. GTT of the female (A) and male offspring (B) at 10 weeks of age during RD and after 5–6 weeks of HFD. Black circles (•)  =  Control offspring on RD, open circles (○)  =  Metformin offspring on RD, Black triangles (▴)  =  Control offspring on HFD, open triangles (Δ)  =  Metformin offspring on HFD. n = 12–16. AUC values of the GTT of the female (C) and male (D) offspring. ** = P<0.01 and *** = P<0.001 by 2-way RM-ANOVA and Sidak's multiple comparisons test. P-value of interaction 0.063 in C and <0.05 in D.

Prenatal metformin treatment did not have a significant effect on the glucose tolerance in either gender measured during the RD at 10 weeks of age (Fig. 6A–B). Six weeks of HFD led to higher blood glucose values in GTT (Fig. 6A–B), however, glucose tolerance was impaired significantly only in the control offspring (Fig. 6C–D). Thus, prenatal exposure to metformin prevented the diet-induced impairment of the glucose tolerance in both genders. ΔAUC^0–90 min (mmol/l)^ from RD to HFD was 120±37 vs. 36±25 (P = 0.063) in the female offspring and 364±74 vs. 123±66 (P<0.05) in the male offspring.

### Serum adipokines, lipids and blood glucose of the offspring

Serum leptin and resistin were quantified from the female and male offspring during the RD (10 weeks of age, n = 7–8). Of note, leptin level of metformin exposed male offspring was decreased (P = 0.051). Leptin levels were not changed in the metformin exposed female offspring (S1A–B Fig.). Resistin, an indicator of insulin resistance in mice [32], was unaffected in both genders at this time point (data not shown).

At the end of the study, the offspring were subjected to two doses of metformin. The glucose lowering effect of the acute metformin was retained in the female offspring (P<0.05, post-treatment; S2 Table) and the male offspring tended to response in a similar way (P = 0.12, post-treatment; S3 Table). Analyses of the serum showed that the metformin exposed male offspring had decreased level of circulating triglycerides (P<0.05, prenatal treatment; S3 Table) and total cholesterol (P<0.05; S3 Table). There was no change in the circulating leptin, insulin and resistin levels. Female offspring exposed prenatally to metformin had decreased level of leptin which was in line with the decreased adiposity (P<0.05; S2 Table). Other serum parameters were not affected in the female offspring.

### Prenatal metformin exposure in neonatal hepatic and SAT pathways of metabolism

**Figure 7:**
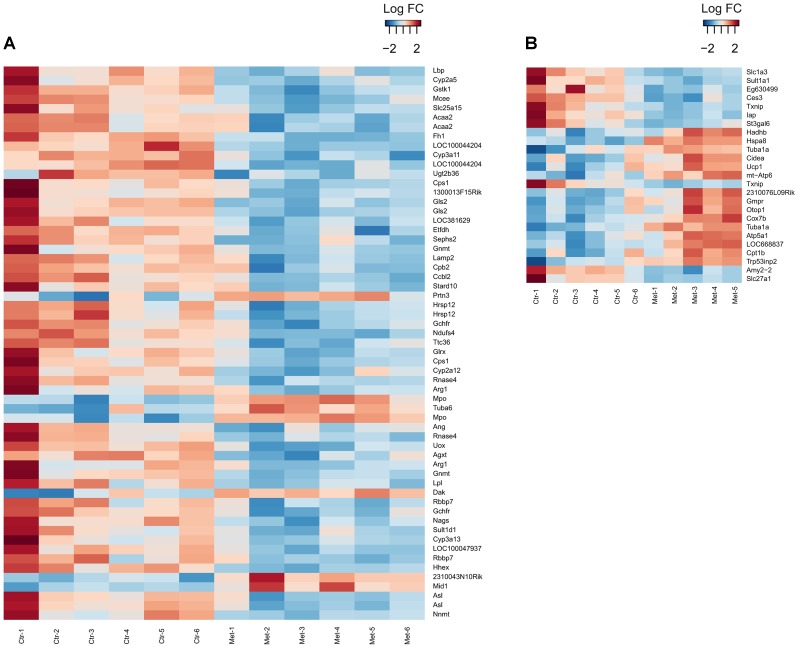
Heatmaps of the liver and SAT gene expression. Hepatic gene expression profile of vehicle and metformin exposed male offspring at the age of 4 days, n = 6 in both groups. Genes with adjusted P-value <0.05 and LogFC cut-off ≥0.5 are shown (A). SAT gene expression profile of vehicle and metformin exposed male offspring at the age of 4 days, n = 6 in the control offspring, n = 5 in the metformin offspring. Genes with adjusted P-value <0.01 and LogFC cut-off ≥1 are shown (B).

To assess the global transcriptome change, microarray profiling was performed on the neonatal liver and SAT derived from the metformin and vehicle exposed 4-day old male offspring. The male offspring were studied due to feasibility. Overall, the gene expression level changes were more pronounced in the SAT as there were 1009 unique differentially expressed genes in the SAT compared to 165 genes in the liver (Fig. 7A–B, S4–S5 Tables).

**Figure 8:**
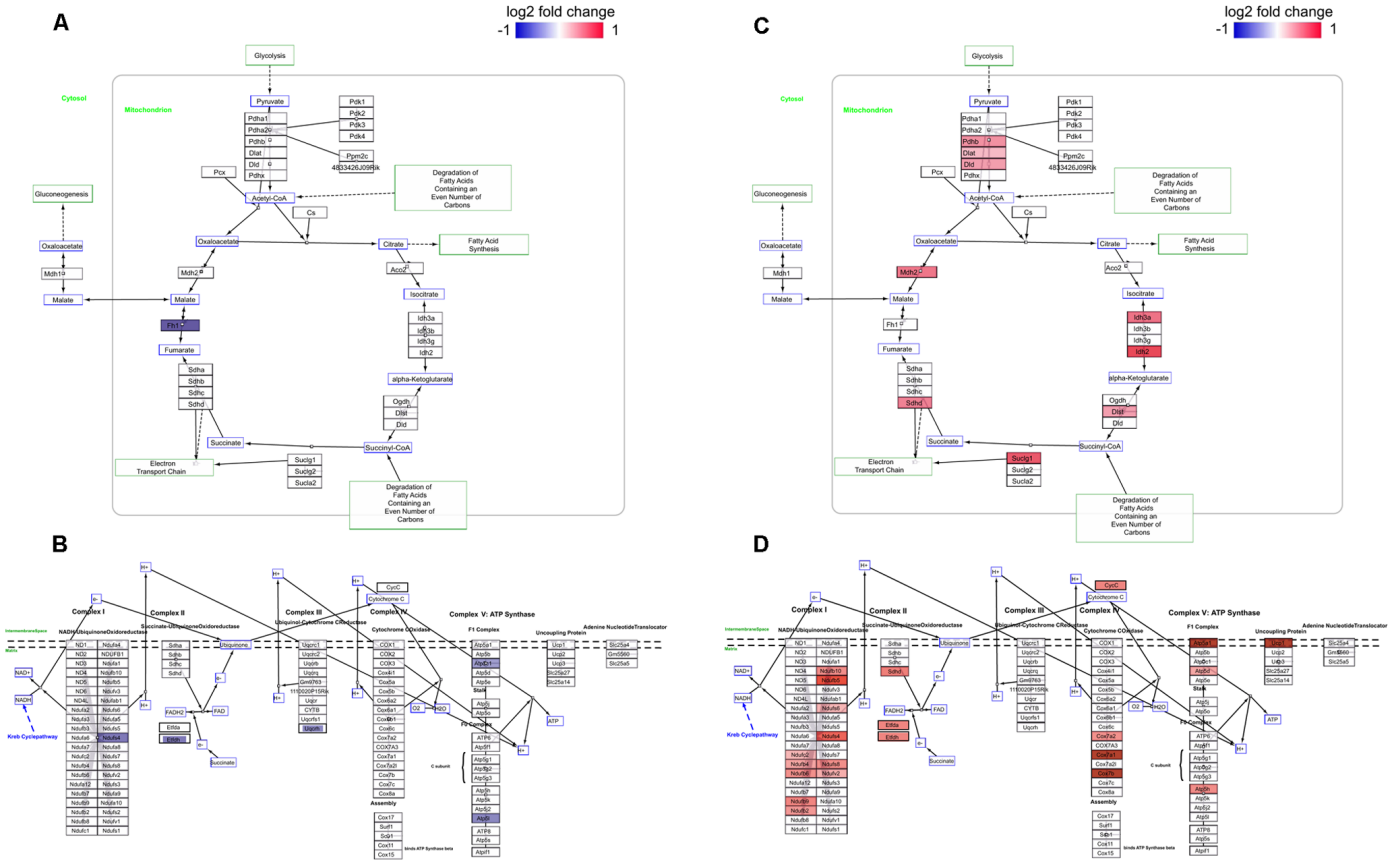
Prenatal metformin exposure in neonatal TCA and ETC pathways. Differentially (P<0.05) expressed genes in TCA cycle and ETC in the liver (A–B) and SAT (C–D) in response to prenatal metformin exposure are shown coloured.

In order to expose the pathways affected by the transcriptome change, enrichment analyses were performed using curated pathway databases KEGG and REACTOME (hypergeometric test, adjusted P-value <0.05). The pathways enriched in both tissues were: The citric acid cycle and respiratory electron transport and respiratory electron transport, ATP synthesis by chemiosmotic coupling, and heat production by uncoupling proteins (REACTOME) and Valine, leucine and isoleucine degradation (KEGG). Furthermore in SAT, pathways associated to immune responses; Antigen Presentation: Folding, assembly and peptide loading of class I MHC, Class I MHC mediated antigen processing & presentation and Antigen processing-Cross presentation were enriched in the metformin group (REACTOME). All the enriched pathways (P<0.05) are presented in S6 Table. Moreover, due to the vast amount of genes detected related to TCA and ETC pathway and based on previously reported effects of metformin on the cellular respiration and ATP production [7], [33], illustrations of metformin targeted genes in the aforementioned pathways are provided in Fig. 8A–D and listing of individual genes in S7–S8 Tables.

**Figure 9:**
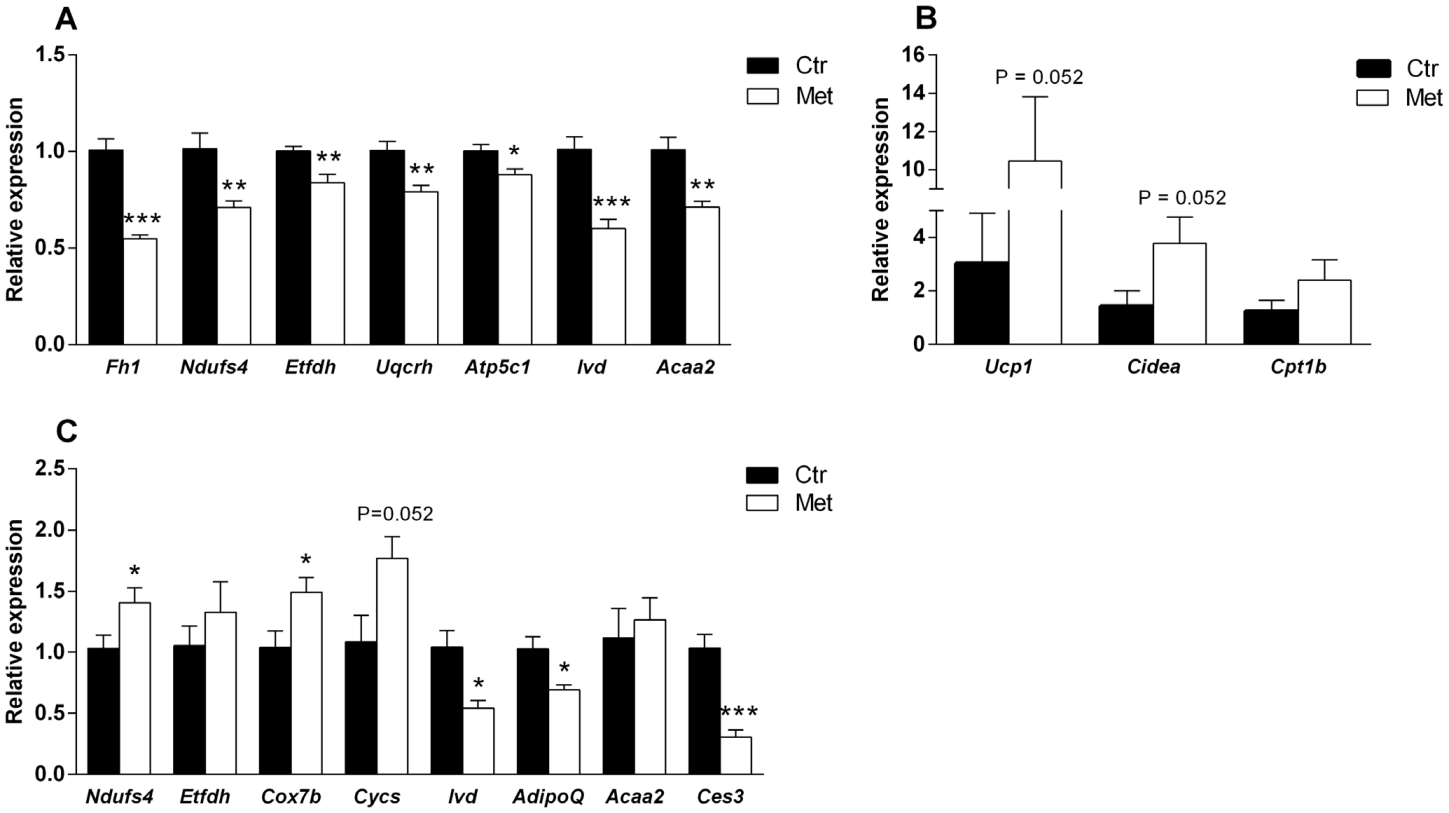
qPCR validation on the microarray-based gene expression profile in the neonatal liver and SAT. The expression of *Fh1* (fumarate hydratase 1), *Ndufs4* (NADH dehydrogenase (ubiquinone) Fe-S protein 4), *Etfdh* (electron transferring flavoprotein, dehydrogenase), *Uqcrh* (ubiquinol-cytochrome c reductase hinge protein), *Atp5c1* (ATP synthase, H^+^transporting, mitochondrial F1 complex, gamma polypeptide 1), *Ivd* (isovaleryl coenzyme A dehydrogenase) and *Acaa2* (acetyl-Coenzyme A acyltransferase 2 (mitochondrial 3-oxoacyl-Coenzyme A thiolase)) in the neonatal liver (A) by qPCR. n = 6 in both groups. * = P<0.05, ** = P<0.01 and *** = P<0.001. The expression of *Ucp1* (uncoupling protein 1), *Cidea* (cell death-inducing DNA fragmentation factor, alpha subunit-like effector A), *Cpt1b* (carnitine palmitoyltransferase 1b) (B), *Ndufs4* (NADH dehydrogenase (ubiquinone) Fe-S protein 4), *Etfdh* (electron transferring flavoprotein, dehydrogenase), *Cox7b* (cytochrome c oxidase subunit VIIb), *Cycs* (cytochrome c), *Ivd* (isovaleryl coenzyme A dehydrogenase), *AdipoQ* (adiponectin), *Acaa2* (acetyl-Coenzyme A acyltransferase 2 (mitochondrial 3-oxoacyl-Coenzyme A thiolase)) and *Ces3* (carboxylesterase 1D) in the neonatal SAT (C) by qPCR. n = 5 in Met and 6 in Ctr group. * = P<0.05 and *** = P<0.001 by Student's t-test or Mann-Whitney test. *Ucp1, Cidea* and *Cpt1b* presented separately due to a different scale in the expression levels.

The results of the pathway analyses support the physiological changes in the metabolic phenotype, and can be used further to study the underlying regulatory mechanism. Several genes of metabolism were validated by qPCR. Related to TCA and ETC pathway, the expression of *Fh1, Ndufs4* (complex I), *Etfdh* (mitochondrial electron transfer flavoprotein complex), *Uqcrh* (complex III) and *Atp5c1* (ATP synthase) were down-regulated in the liver in response to metformin exposure (Fig. 9A) indicating a down-shift in the ATP production. In the SAT, *Ndufs4*, *Cox7b* (complex IV), Cycs* (cytochrome c) together with Ucp1* were up-regulated (*P-value 0.052, Fig. 9B–C). Additionally, the expression of *Cidea* was up-regulated (P = 0.052) and *AdipoQ* down-regulated in the SAT in the metformin exposed offspring (Fig. 9B–C). The combined effects in the SAT suggest a phenotypic change towards brown adipose tissue. This was assessed by a comparison (P<0.001) of a set of browning-associated genes (*Prdm16, Ucp1, CIDEA, Cox8b, GLUT4, Ppargca1, Elovl3, Fabp4, AdipoQ*) according to Seale et al. [31].

Concerning the valine, leucine and isoleucine degradation pathway (i.e. branched-chain amino acid (BCAA) metabolism), *Ivd* (isovaleryl coenzyme A dehydrogenase) and *Acaa2* (acetyl-Coenzyme A acyltransferase 2) showed down-regulation in the liver (Fig. 9A). *Ivd* was also down-regulated in the SAT (Fig. 9C). Furthermore, microarray profiling showed *Bcat2* and *Bckdha* to be down-regulated in the SAT (not validated by qPCR) indicating the engagement of BCAA metabolism in the processes at neonatal age.

Finally, *Ces3* (also *Ces1d, TGH*) was strongly down-regulated in response to metformin in the SAT (Fig. 9C). *Ces3* codes for carboxylesterase 1D, which has recently been connected to obesity, diabetes and lipid metabolism [34], [35]. In addition to this, *Txnip* (thioredoxin-interacting protein; not validated by qPCR) was down-regulated in the metformin offspring. *Txnip* has been shown to inhibit cellular glucose uptake [36] and convey hyperglycemia induced inflammation [37].

## Discussion

Diabetic environment *in utero* predisposes fetuses and newborn to macrosomia, hypoglycemia and other perinatal complications [10], [14]. Importantly, it also increases the risk for later metabolic disorders in the offspring [11], [12]. Thus, it is important to normalise the maternal metabolism in diabetic pregnancies with diet, insulin or, as recent studies suggest, with metformin [18], [20]. Our previous study showed that when metformin is administered to dams on a regular diet, the offspring are predisposed to diet-induced obesity [24]. Furthermore, the male offspring have impaired glucose metabolism on HFD. With the current mouse model we demonstrated that prenatal metformin exposure has largely a protective effect on the metabolic phenotype of the offspring during HFD. This was more prominently seen in the female offspring by a decrease in the adipose tissue accumulation and in the prevention of HFD-induced impairment in glucose tolerance in both genders. Moreover, microarray profiling revealed major changes in the gene expression pattern at neonatal age with possible implications to the phenotype observed during HFD.

At present, there are several clinical studies that report the efficacy and safety of metformin treatment in GDM and pregnancies complicated by PCOS [16]–[18], [20], [21]. However, there are few experimental set-ups which include the long-term follow-up of the offspring. Rowan et al. have reported that when women with GDM were treated with metformin, alone or in combination with insulin, the offspring at two years had more subcutaneous fat without the expense of the total amount of fat compared to those exposed only to insulin [38]. These changes in the fat distribution were discussed to provide a protection against later accumulation of ectopic fat. However, in humans, this hypothesis cannot be validated in adult life for a long time.

Rodent models have been used extensively to investigate the effects of maternal obesity, obesogenic diet and hyperglycemia to the metabolic profile of the offspring [22]. The benefit of rodent studies rely on feasible performance and study designs which include several approaches aiming at a comprehensive characterisation of a phenomenon. However, it must be noted that the metabolism and the external environment in rodents and humans (e.g. during pregnancy) are also different and all the effects observed during the course of the studies may not be translatable. Nevertheless, the value of the studies resides in the characterisation of target organs and systems affected. In the present study, we used a metabolic model (pre-conceptual and gestational HFD) characterised by Liang et al. to model human GDM and vasculopathy of the placenta [39]. In our study, fasting blood glucose was significantly elevated in the mice on HFD compared to those on RD indicating that HFD does induce metabolic changes. However, similar to Liang et al., we did not see an effect of the HFD on the body weight before the conception. Female C56BL/6 mice have been shown to be relatively resistant to diet-induced obesity and the effects on the body weight might need a longer HFD period [40].

The dose (300 mg/kg) for metformin administration was selected based on previously published rodent studies [24]–[29]. The dose was well tolerated with no signs of toxicity, and allometrically scales up to human clinical doses of 1.7 g/day to a 70 kg man [24]. Yet, we detected a small but significant decrease in the weight of metformin exposed fetuses at E18.5 (considered as birth weight). This might be attributed to the blood glucose lowering effect of metformin that was observed in the pregnant dams after metformin administration. Other major changes in the phenotype of the offspring were not observed during the RD phase as assessed by the body weight gain, food intake, adipokine measurements at the age of four weeks (male offspring), and glucose tolerance at the age of 10 weeks. One interesting finding during the RD phase at 10 weeks of age was however decreased serum leptin levels in the metformin exposed male offspring without a significant difference in the body weight. Body composition was not measured at this time point but the result could indicate lower amount of adipose tissue. It seems that the phenotypic differences between control and metformin exposed offspring do not manifest without a clear metabolic challenge. During the HFD phase, a preventive effect of prenatal metformin exposure to diet-induced impairment of glucose tolerance was observed. As there were no changes in the insulin levels at the end of the study, we hypothesise that the effects on the glucose metabolism may be mediated by improved insulin sensitivity. However, we did not perform insulin sensitivity tests and our *GLUT4* expression data in eWAT of the male offspring does not demonstrate significant effects on the receptor expression in the adipose tissue (S2 Fig.).

A study of the long-term effects of metformin in a metabolic model was recently published by Tong et al [29]. In their research, the offspring were exposed to metformin during the pregnancy and lactation as we administered metformin only during the gestation in order to investigate the prenatal effects of metformin exposure. Nevertheless, similar to our current data Tong et al. describe a largely improved metabolic profile of the metformin exposed offspring. Specifically, there was an increased mitochondrial content and higher oxidative phosphorylation (OXPHOS) capacity of the muscle. Our study on the other hand extended the knowledge of prenatal metformin exposure on the adipose tissue accumulation and improved metabolism. Moreover, the cues for the altered phenotype were found from the neonatal gene expression pattern.

To study the neonatal gene expression pattern and potential metabolic pathways responsible for the effects on the phenotype, we performed microarray analyses of the liver and SAT of the 4-day old male offspring. Perinatal time frame is a critical period for development in mice and provides a sensitive time window for environmental influences [41]. Studies exploring the mechanisms of energy balance show that giving a suitable stimulus (e.g. leptin) during the perinatal period can have persistent effects on the metabolic phenotype, i.e. programming [42], [43]. Programming occurs most likely *via* epigenetic modulation of the gene expression [44]. Thus, we were prompted to investigate particularly the early changes in the gene expression levels. Whether the changes observed at the neonatal age are permanent, remains unclear and require further studies.

Liver is a target tissue of metformin as it is the principal tissue delivering glucose to the body. Interestingly in our data, several ETC pathway genes were down-regulated in the liver in response to metformin. Effects were detected e.g. in the down-regulated expression of genes associated with ETC complex I (*Ndufs4*), complex III (*Uqcrh*) and ATP synthase (*Atp5c1*). Previously, the inhibitory effects of metformin on the complex I of the ETC have been reported in a few individual studies [33], [45]. Moreover, it has been demonstrated that metformin affects the rate of gluconeogenesis by decreasing cellular ATP levels in the liver [7]. Furthermore, the level of mitochondrial functionality in metabolic disorders is a matter of great interest [46]. Although defects in the cellular respiration have been held as a cause for impaired metabolism, a recent mouse model of mitochondrial OXPHOS deficiency (*AIF* KO) shows that a reduction in OXPHOS protects against metabolic disturbances [47]. Our analyses support the effects of metformin on the ETC complexes and indicate further a therapeutic effect of metformin in the fetal liver which might have long-term effects in our model.

In contrast to the liver, adipose tissue is not considered as a primary target tissue of metformin. However, adipocytes have an essential role in major metabolic disorders and based on our previous and present results of adipose tissue accumulation, we considered vital to investigate the gene expression pattern of the adipose tissue. In the SAT, there were several pathways of metabolism affected by prenatal metformin exposure (S6 Table). In contrast to the data obtained from the liver, the effects on the ETC were the opposite. Genes associated to the mitochondrial ETC pathway (*Ndufs4*, *Cycs, Cox7b* and *Ucp1)* were up-regulated by metformin exposure. Moreover, there was a strong link in our dataset with what is previously known about the “browning” of white adipocytes [31]. Recent reports have identified a special population of white adipocytes able to transform to a brown-like phenotype under suitable stimuli such as cold exposure, pharmacological β_3_ -adrenergic stimulation and anti-diabetic thiazolidinedione treatment [31], [48]–[52]. A mouse model of white and brown fat specific *Ucp1* expression shows that the induction of *Ucp1* together with other mitochondrial genes in white adipose tissue attributes to a lean phenotype [53]. Studies have shown that this type of phenotypic shift may also lead to resistance to diet-induced obesity [31], [54] as can be indicated also in our study. Body composition measurement after six weeks of HFD at adulthood revealed that prenatal metformin exposure resulted in a decreased amount of adipose tissue, especially in the female offspring.

Microarray data revealed also that a novel obesity-associated gene, *Ces3*, was down-regulated by metformin exposure in the neonatal adipose tissue. *Ces3* is a regulator of lipolysis and was recently found to promote lipid storage in adipocytes [34]. Interestingly, we observed that metformin exposed female offspring have a higher percentage of smaller and a minor percentage of larger adipocytes at the end of the HFD phase. Furthermore, the analysis of the number of the adipocytes in gWAT depot showed a strong tendency to hypoplasia in the metformin offspring. The down-regulation of *Ces3* may be a link in the observed hypotrophy and hypoplasia although further studies are needed to verify this.

Given all our data on the effects of metformin on the dams and offspring, it is most probable that the effects are both direct and indirect. The fact that metformin lowered the blood glucose of the HFD dams is a mark of beneficial effects on the maternal glucose metabolism and it contributes also to the growth environment of the fetuses. However, as metformin readily crosses the placenta [24], the direct effects are also undoubtedly present in the fetus. It must be noted that the distinction between these two mechanisms is utterly challenging with experimental set-ups.

In summary, the present study provides evidence that prenatal exposure to metformin in the maternal HFD mouse model has beneficial influences on the glucose tolerance, body weight development and fat mass accumulation in the offspring during HFD. Furthermore, metformin exposure affects the expression of several metabolic genes and pathways at neonatal age indicating that it modifies a large network of metabolic processes like the ETC pathway. Finally, together with our previous data [24] it seems evident that the maternal metabolic status during gestation has a crucial role in determining how the prenatal metformin exposure translates to the phenotype of the offspring.

## Supporting Information

S1 Fig Offspring serum leptin during RD. Leptin levels of the female (A) and male (B) offspring at the end of the RD phase at 10 weeks of age. n = 7–8. P = 0.054 by Mann-Whitney test.
(PDF)

S2 Fig *GLUT4* mRNA expression in the eWAT at the end of the HFD phase. +/− denotes whether the mice were given an acute metformin dosage (2×300 mg/kg, p.o.), n = 5–8. Data expressed as mean ±SEM.
(PDF)

S1 Table Sequences (5′–>3′) of the primer pairs used for qPCR.
(PDF)

S2 Table Blood glucose, lipids and adipokines of the female offspring. P<0.05; prenatal treatment effect by 2-way ANOVA. +/− denotes whether the mice were given an acute metformin dosage (2×300 mg/kg, p.o.). n = 3–8. Data expressed as mean ±SEM.
(PDF)

S3 Table Blood glucose, lipids and adipokines of the male offspring. P<0.05; prenatal treatment effect by 2-way ANOVA. +/− denotes whether the mice were given an acute metformin dosage (2×300 mg/kg, p.o.). n = 5–8. Data expressed as mean ±SEM.
(PDF)

S4 Table Differentially (P<0.05) expressed genes in the liver in response to prenatal metformin exposure. In the liver, the expression of 165 unique genes was changed significantly. Table contains all the probes/gene (number of rows 185) shown from the highest up-regulation to the lowest down-regulation, n = 6 in both groups. LogFC  =  fold change in a logarithmic scale.
(PDF)

S5 Table Differentially (P<0.05) expressed genes in the SAT in response to prenatal metformin exposure. In the SAT, the expression of 1009 unique genes was changed significantly. Table contains all the probes/gene (number of rows 1082) shown from the highest up-regulation to the lowest down-regulation, n = 5–6. LogFC  =  fold change in a logarithmic scale.
(PDF)

S6 Table Enriched pathways in the liver and SAT according to the hypergeometric test. q-value equals to P-value.
(PDF)

S7 Table Genes associated to REACTOME pathway citric acid (TCA) cycle and respiratory electron transport (ETC).
(PDF)

S8 Table Genes associated to REACTOME pathway respiratory electron transport, ATP synthesis by chemiosmotic coupling, and heat production by uncoupling proteins.
(PDF)
