# Supplementary figures and images for: Prenatal Metformin Exposure in a Maternal High Fat Diet Mouse Model Alters the Transcriptome and Modifies the Metabolic Responses of the Offspring
Source: PLoS One. 2014 Dec 26;9(12):e115778. doi: 10.1371/journal.pone.0115778 (PMC4277397; doi:10.1371/journal.pone.0115778)

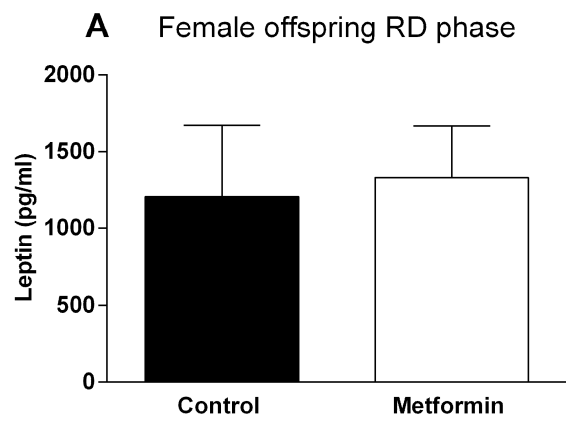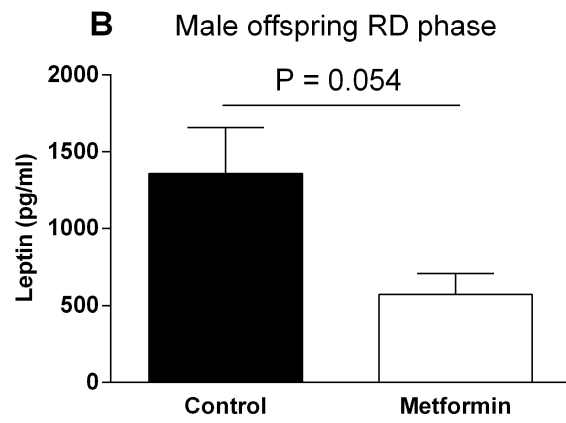

Supplement: S1 Fig — Offspring serum leptin during RD. Leptin levels of the female (A) and male (B) offspring at the end of the RD phase at 10 weeks of age. n = 7–8. P = 0.054 by Mann-Whitney test. (PDF) [file pone.0115778.s001.pdf]

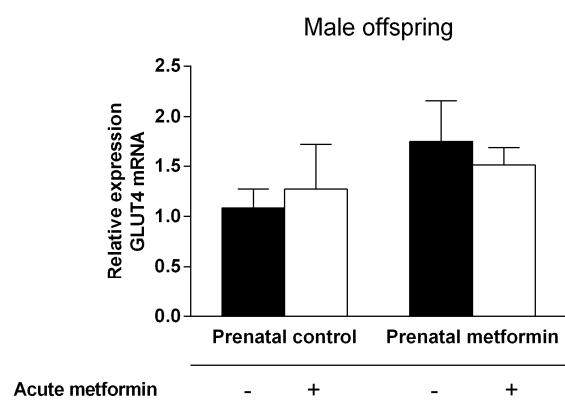

Supplement: S2 Fig — GLUT4 mRNA expression in the eWAT at the end of the HFD phase. +/− denotes whether the mice were given an acute metformin dosage (2×300 mg/kg, p.o.), n = 5–8. Data expressed as mean ±SEM. (PDF) [file pone.0115778.s002.pdf]
