# Supplementary material for: Prenatal Metformin Exposure in a Maternal High Fat Diet Mouse Model Alters the Transcriptome and Modifies the Metabolic Responses of the Offspring
Source: PLoS One. 2014 Dec 26;9(12):e115778. doi: 10.1371/journal.pone.0115778 (PMC4277397; doi:10.1371/journal.pone.0115778)
Supplement: S1 Table — Sequences (5′–>3′) of the primer pairs used for qPCR. (PDF) [file pone.0115778.s003.pdf]

**Table S1. Sequences (5' -> 3') of the primer pairs used for qPCR.**

| Gene | Gene Accession | Forward (5' -> 3') | Reverse (5' -> 3') |
|---|---|---|---|
| Acaa2 | NM_177470.3 | CTGCTACGAGGTGTGTTCATC | AGCTCTGCATGACATTGCCC |
| AdipoQ | NM_009605.4 | AGGGCTCAGGATGCTACTGT | CACAAGTTCCCTTGGGTGGA |
| Atp5c1 | NM_020615.4 | CAAGGCTCCACCATGTTCTC | TTCAGAGTTGCCATGTTTCG |
| Ces3 | NM_053200.2 | TGGTATTTGGTGTCCCATCA | GCTTGGGCGATACTCAAACT |
| Cidea | NM_007702.2 | GCCGTGTTAAGGAATCTGCTG | TGCTCTTCTGTATCGCCCAGT |
| Cox7b | NM_025379.2 | TGTTGCCCTTAGCCAAAAAC | AAGATGGCTCCACCTGCTAA |
| Cpt1b | NM_009948.2 | GAGTGACTGGTGGGAAGAATATG | GCTGCTTGCACATTTGTGTT |
| Cycs | NM_007808.4 | GGACGTCTGTCTTCGAGTCC | ACTGGGCACACTTCTGAACA |
| Etfdh | NM_025794.2 | GTGCGACTAACCAAGCTGTC | GGATGAACAGTGTAGTGAGTGG |
| Fh1 | NM_010209.2 | GAATGGCAAGCCAAAATTCCTT | CGTTCCGTAGCACCTCCAATCTT |
| Glut4 | NM_009204.2 | GACGGACACTCCATCTGTTG | GCCACGATGGAGACATAGC |
| Ivd | NM_019826.3 | CACTCGATATTGCCTGTGGA | TGATCAATCTCTTGGGCCTT |
| Ndufs4 | NM_010887.2 | CTGCCGTTTCCGTCTGTAGAG | TGTTATTGCGAGCAGGAACAAA |
| Rps29 | NM_009093.2 | ATGGGTCACCAGCAGCTCTA | AGCCTATGTCCTTCGCGTACT |
| Ucp1 | NM_009463.3 | ACTGCCACACCTCCAGTCATT | CTTTGCCTCACTCAGGATTGG |
| Uqcrh | NM_025641.3 | GTGGACCCCCTAACAACAGTG | CGGGAAGACACGCGATTATCA |
