# Supplementary material for: Prenatal Metformin Exposure in a Maternal High Fat Diet Mouse Model Alters the Transcriptome and Modifies the Metabolic Responses of the Offspring
Source: PLoS One. 2014 Dec 26;9(12):e115778. doi: 10.1371/journal.pone.0115778 (PMC4277397; doi:10.1371/journal.pone.0115778)
Supplement: S2 Table — Blood glucose, lipids and adipokines of the female offspring. P<0.05; prenatal treatment effect by 2-way ANOVA. +/− denotes whether the mice were given an acute metformin dosage (2×300 mg/kg, p.o.). n = 3–8. Data expressed as mean ±SEM. (PDF) [file pone.0115778.s004.pdf]

Table S2. Blood glucose, lipids and adipokines of the female offspring. $P < 0.05$; prenatal treatment effect by 2-way ANOVA. +/- denotes whether the mice were given an acute metformin dosage (2 X 300 mg/kg, p.o.). n = 3 - 8. Data expressed as mean ± SEM.

		Prenatal group				
		Ctr	Ctr	Met	Met	P-value *Prenatal treatment*
Acute metformin		-	+	-	+	
	Glucose (mmol/l)	8.7 ± 0.8	7.0 ± 0.5	9.6 ± 0.8	7.9 ± 0.5	*NS*[*)]
	Trigly (mg/ml)	0.54 ± 0.22	0.62 ± 0.1	0.63 ± 0.16	0.54 ± 0.13	*NS*
	Cholesterol (mM)	0.56 ± 0.13	0.70 ± 0.10	0.47 ± 0.05	0.52 ± 0.07	*NS*
	NEFA (mmol/l)	0.30 ± 0.04	0.27 ± 0.04	0.29 ± 0.06	0.24 ± 0.03	*NS*
	Leptin (pg/ml)	8473 ± 1966	7618 ± 1338	5775 ± 1619	3599 ± 1288	< 0.05
	Insulin (pg/ml)	268 ± 119	308 ± 188	335 ± 86	621 ± 235	*NS*
	Resistin (pg/ml)	2000 ± 387	1713 ± 202	2116 ± 341	1745 ± 215	*NS*

[*)] Acute metformin $P < 0.05$
