# Supplementary material for: Prenatal Metformin Exposure in a Maternal High Fat Diet Mouse Model Alters the Transcriptome and Modifies the Metabolic Responses of the Offspring
Source: PLoS One. 2014 Dec 26;9(12):e115778. doi: 10.1371/journal.pone.0115778 (PMC4277397; doi:10.1371/journal.pone.0115778)
Supplement: S3 Table — Blood glucose, lipids and adipokines of the male offspring. P<0.05; prenatal treatment effect by 2-way ANOVA. +/− denotes whether the mice were given an acute metformin dosage (2×300 mg/kg, p.o.). n = 5–8. Data expressed as mean ±SEM. (PDF) [file pone.0115778.s005.pdf]

Table S3. Blood glucose, lipids and adipokines of the male offspring. $P < 0.05$; prenatal treatment effect by 2-way ANOVA. +/- denotes whether the mice were given an acute metformin dosage (2 X 300 mg/kg, p.o.). n = 5 - 8. Data expressed as mean ± SEM.

		Prenatal group				
		Ctr	Ctr	Met	Met	P-value *Prenatal treatment*
Acute metformin		-	+	-	+	
	Glucose (mmol/l)	8.3 ± 0.8	7.8 ± 0.9	10.3 ± 0.5	8.4±0.6	*NS*[*)]
	Trigly (mg/ml)	0.82 ± 0.20	0.80 ± 0.10	0.47 ± 0.08	0.48 ± 0.13	< 0.05
	Cholesterol (mM)	1.76 ± 0.19	1.80 ± 0.14	1.48 ± 0.14	1.29 ± 0.11	< 0.05
	NEFA (mmol/l)	0.26 ± 0.03	0.28 ± 0.02	0.26 ± 0.03	0.26 ±0.04	*NS*
	Leptin (pg/ml)	13729 ± 4481	13720 ± 3369	14388 ± 2426	7253±2319	*NS*
	Insulin (pg/ml)	474 ± 61	465 ± 62	423 ± 96	359±70	*NS*
	Resistin (pg/ml)	1583 ± 250	1698 ± 229	2283 ± 402	1584±176	*NS*

[*)] Acute metformin P = 0.12
