# Supplementary material for: Prenatal Metformin Exposure in a Maternal High Fat Diet Mouse Model Alters the Transcriptome and Modifies the Metabolic Responses of the Offspring
Source: PLoS One. 2014 Dec 26;9(12):e115778. doi: 10.1371/journal.pone.0115778 (PMC4277397; doi:10.1371/journal.pone.0115778)
Supplement: S4 Table — Differentially (P<0.05) expressed genes in the liver in response to prenatal metformin exposure. In the liver, the expression of 165 unique genes was changed significantly. Table contains all the probes/gene (number of rows 185) shown from the highest up-regulation to the lowest down-regulation, n = 6 in both groups. LogFC = fold change in a logarithmic scale. (PDF) [file pone.0115778.s006.pdf]

Table S4. Differentially (P < 0.05) expressed genes in the liver in response to prenatal metformin exposure. In the liver, the expression of 165 unique genes was changed significantly. Table contains all the probes/gene (number of rows 185) shown from the highest up-regulation to the lowest down-regulation, n = 6 in both groups. LogFC = fold change in a logarithmic scale.

GENE Entrez	GENE Symbol	LogFC	Adj. P-value
17318	Mid1	1.22	0.011
225913	Dak	1.04	0.005
17523	Mpo	0.78	0.000
17523	Mpo	0.70	0.001
66961	2310043N10Rik	0.69	0.007
19152	Prtn3	0.51	0.004
217125	Samd14	0.50	0.016
100045999	LOC100045999	0.48	0.047
107503	Atf5	0.47	0.018
23925	Kel	0.47	0.028
19044	Ppox	0.44	0.012
17002	Ltf	0.43	0.043
15384	Hnrnpab	0.43	0.047
100047651	LOC100047651	0.43	0.018
11684	Alox12	0.42	0.022
103711	Pnpo	0.39	0.025
17523	Mpo	0.36	0.001
50701	Ela2	0.34	0.030
252870	Usp7	0.34	0.017
16399	Itga2b	0.34	0.004
19185	Psmd4	0.34	0.037
71643	4930422G04Rik	0.32	0.006
235606	Apeh	0.31	0.038
70026	Bzrpl1	0.30	0.047
18770	Pklr	0.28	0.012
16534	Kcnn4	0.28	0.025
20637	Snrp70	0.27	0.040
231830	Micall2	0.25	0.044
71941	2310051N18Rik	0.24	0.027
108052	Slc14a1	0.22	0.006
27205	Podxl	0.22	0.038
66532	2210417D09Rik	0.22	0.015
239552	Apol2	0.22	0.016
214901	Chtf18	0.22	0.047
67655	Ctdp1	0.21	0.018
22594	Xrcc1	0.20	0.024
100047674	LOC100047674	0.20	0.034
217353	Tmc6	0.19	0.040
67112	Fgf22	0.19	0.028

213988	Tnrc6b	0.19	0.007
68925	Rpap1	0.18	0.024
106205	Zc3h7a	0.17	0.046
442827	9830134C10Rik	0.17	0.017
59035	Carm1	0.17	0.044
50997	Mpp2	0.17	0.047
241525	Ypel4	0.16	0.003
21825	Thbs1	0.15	0.028
68964	1500010J02Rik	0.15	0.047
170676	Peg10	0.14	0.029
240753	Plekha6	0.12	0.018
12695	Inadl	0.11	0.039
69585	Hfe2	-0.12	0.042
211401	Mtss1	-0.12	0.048
100609	Nsun5	-0.12	0.024
83490	Pik3ap1	-0.14	0.027
97487	Cmtm4	-0.14	0.031
20913	Stxbp4	-0.16	0.014
14287	Fpgs	-0.16	0.032
75462	1700001C19Rik	-0.17	0.034
330463	Zfp78	-0.17	0.018
211389	Suox	-0.18	0.040
74931	4930481A15Rik	-0.18	0.025
381534	Ube2u	-0.18	0.043
67674	0610038D11Rik	-0.18	0.005
71562	Afmid	-0.18	0.010
234878	BC021891	-0.19	0.010
223920	Soat2	-0.19	0.017
27215	Azi2	-0.19	0.008
69770	1600002K03Rik	-0.22	0.017
98845	Eps8l2	-0.22	0.028
24059	Slco2a1	-0.22	0.048
214424	Parp16	-0.23	0.010
66129	1110018J18Rik	-0.24	0.048
194433	Olfr707	-0.24	0.012
71336	Rbks	-0.25	0.019
216871	Gltpd2	-0.25	0.003
56350	Arl3	-0.26	0.023
68338	Golt1a	-0.26	0.039
30956	Aass	-0.26	0.040
68347	0610011F06Rik	-0.27	0.011
56629	Dnase2b	-0.27	0.003
85031	Pla1a	-0.27	0.000
66487	2010107H07Rik	-0.27	0.030
76429	2310007H09Rik	-0.27	0.047

85031	Pla1a	-0.28	0.023
94279	Sfxn2	-0.28	0.005
100034251	OTTMUSG00000000971	-0.28	0.021
20751	Spr	-0.28	0.022
50702	Cfhr1	-0.29	0.006
69745	Pold4	-0.29	0.047
68312	Gstm7	-0.29	0.014
50765	Trfr2	-0.29	0.046
71562	Afmid	-0.29	0.004
71562	Afmid	-0.30	0.001
381045	Ccdc58	-0.31	0.030
66333	Aqp11	-0.31	0.018
103149	Upb1	-0.31	0.030
231903	Prhoxnb	-0.31	0.027
76257	Slc38a3	-0.31	0.042
17175	Masp2	-0.32	0.044
66412	Arrdc4	-0.32	0.035
16621	Klkb1	-0.32	0.024
76654	Upp2	-0.33	0.022
93747	Echs1	-0.34	0.046
15160	Serpind1	-0.36	0.032
71760	Agxt2l1	-0.37	0.047
76654	Upp2	-0.37	0.007
223267	a2ld1	-0.38	0.007
66847	Hint3	-0.38	0.037
94279	Sfxn2	-0.38	0.030
66847	Hint3	-0.39	0.032
18105	Nqo2	-0.39	0.047
67732	Iah1	-0.39	0.027
11949	Atp5c1	-0.39	0.000
268860	Abat	-0.40	0.001
17195	Mbl2	-0.40	0.011
56357	Ivd	-0.41	0.013
64385	Cyp4f14	-0.41	0.047
70031	Cmtm8	-0.41	0.008
15473	Hrsp12	-0.42	0.008
107747	Aldh1l1	-0.42	0.026
58992	F12	-0.43	0.047
18105	Nqo2	-0.44	0.005
237320	Aldh8a1	-0.44	0.010
56373	Cpb2	-0.44	0.033
11607	Agtr1a	-0.45	0.041
382044	Ces1b	-0.45	0.042
27409	Abcg5	-0.45	0.047
67880	Dcxr	-0.45	0.017

654426	LOC654426	-0.45	0.025
19301	Pxmp2	-0.45	0.049
54151	Cyhr1	-0.45	0.025
30057	Timm8b	-0.45	0.006
18478	Pah	-0.46	0.032
66576	Uqcrh	-0.46	0.017
67880	Dcxr	-0.46	0.001
100047762	LOC100047762	-0.47	0.004
217214	Nags	-0.47	0.002
27425	Atp5l	-0.48	0.037
69379	C8g	-0.48	0.007
93692	Glrx	-0.50	0.034
100047937	LOC100047937	-0.51	0.016
73724	Mcee	-0.53	0.006
20768	Sephs2	-0.53	0.017
16784	Lamp2	-0.53	0.024
56018	Stard10	-0.53	0.015
56373	Cpb2	-0.54	0.013
66841	Etfdh	-0.55	0.041
17993	Ndufs4	-0.55	0.016
100044204	LOC100044204	-0.56	0.041
13087	Cyp2a5	-0.56	0.005
15473	Hrsp12	-0.56	0.006
217214	Nags	-0.56	0.001
13085	Cyp2a12	-0.56	0.010
11611	Agxt	-0.56	0.003
16803	Lbp	-0.57	0.008
53315	Sult1d1	-0.57	0.006
100044204	LOC100044204	-0.57	0.004
192653	Ttc36	-0.57	0.001
18408	Slc25a15	-0.57	0.011
320415	Gchfr	-0.58	0.000
245688	Rbbp7	-0.60	0.047
18113	Nnmt	-0.60	0.032
22262	Uox	-0.60	0.001
13112	Cyp3a11	-0.62	0.000
320415	Gchfr	-0.62	0.014
15473	Hrsp12	-0.64	0.003
245688	Rbbp7	-0.65	0.045
231396	Ugt2b36	-0.66	0.004
11727	Ang	-0.66	0.017
16956	Lpl	-0.67	0.004
229905	Ccbl2	-0.68	0.036
14194	Fh1	-0.69	0.000
76263	Gstk1	-0.71	0.000

52538	Acaa2	-0.72	0.039
13113	Cyp3a13	-0.73	0.028
15242	Hhex	-0.87	0.000
14711	Gnmt	-0.88	0.005
14711	Gnmt	-0.89	0.004
11846	Arg1	-0.94	0.039
227231	Cps1	-0.98	0.006
216456	Gls2	-1.02	0.000
58809	Rnase4	-1.02	0.039
216456	Gls2	-1.04	0.000
227231	Cps1	-1.04	0.003
