# Supplementary material for: Prenatal Metformin Exposure in a Maternal High Fat Diet Mouse Model Alters the Transcriptome and Modifies the Metabolic Responses of the Offspring
Source: PLoS One. 2014 Dec 26;9(12):e115778. doi: 10.1371/journal.pone.0115778 (PMC4277397; doi:10.1371/journal.pone.0115778)
Supplement: S5 Table — Differentially (P<0.05) expressed genes in the SAT in response to prenatal metformin exposure. In the SAT, the expression of 1009 unique genes was changed significantly. Table contains all the probes/gene (number of rows 1082) shown from the highest up-regulation to the lowest down-regulation, n = 5–6. LogFC = fold change in a logarithmic scale. (PDF) [file pone.0115778.s007.pdf]

Table S5. Differentially (P < 0.05) expressed genes in the SAT in response to prenatal metformin exposure. In the SAT, the expression of 1009 unique genes was changed significantly. Table contains all the probes/gene (number of rows 1082) shown from the highest up-regulation to the lowest down-regulation, n = 5-6. LogFC = fold change in a logarithmic scale.

GENE Entrez	GENE Symbol	LogFC	Adj. P-value
17907	Mylpf	2.56	0.027
22227	Ucp1	2.51	0.001
17907	Mylpf	2.36	0.016
21953	Tnni2	2.07	0.036
14077	Fabp3	1.90	0.017
66402	Sln	1.70	0.046
12683	Cidea	1.63	0.001
21957	Tnnt3	1.59	0.030
17901	Myl1	1.47	0.032
13808	Eno3	1.32	0.043
22142	Tuba1a	1.30	0.005
668837	LOC668837	1.30	0.000
12865	Cox7a1	1.29	0.012
66355	Gmpr	1.21	0.002
12895	Cpt1b	1.18	0.006
22142	Tuba1a	1.18	0.000
66968	2310076L09Rik	1.10	0.001
53318	Pdlim3	1.08	0.024
17294	Mest	1.06	0.038
11464	Actc1	1.06	0.017
11946	Atp5a1	1.05	0.003
14120	Fbp2	1.04	0.019
231086	Hadhb	1.04	0.002
68728	Trp53inp2	1.03	0.001
66142	Cox7b	1.02	0.000
15481	Hspa8	1.02	0.001
17896	Myl4	1.01	0.033
21906	Otop1	1.00	0.004
14571	Gpd2	1.00	0.018
12869	Cox8b	0.98	0.008
67512	Agpat2	0.97	0.022
80888	Hspb8	0.93	0.005
66390	Slmo2	0.90	0.001
269951	Idh2	0.86	0.003
67951	Tubb6	0.86	0.036
1E+08	LOC100048480	0.86	0.002
18655	Pgk1	0.85	0.005
170439	Elovl6	0.85	0.005
27280	Phlda3	0.85	0.009

59011	Myoz1	0.85	0.028
17993	Ndufs4	0.85	0.000
233199	Mybpc2	0.83	0.005
75735	Pank1	0.83	0.010
27280	Phlda3	0.81	0.001
67267	2900010M23Rik	0.80	0.005
66046	Ndufb5	0.80	0.001
52538	Acaa2	0.79	0.010
56451	Suclg1	0.77	0.001
13063	Cycs	0.77	0.006
232370	Clstn3	0.77	0.009
66359	2310005N03Rik	0.75	0.001
17901	Myl1	0.73	0.030
12895	Cpt1b	0.72	0.012
75552	Paqr9	0.71	0.024
14609	Gja1	0.71	0.037
17993	Ndufs4	0.70	0.000
14412	Slc6a13	0.70	0.005
18140	Uhrf1	0.69	0.011
1E+08	LOC100044087	0.69	0.004
65112	Pmepa1	0.68	0.000
66039	D14Ertd449e	0.68	0.015
110842	Etfa	0.67	0.005
15528	Hspe1	0.67	0.006
380773	1810035L17Rik	0.66	0.005
15510	Hspd1	0.66	0.001
632667	LOC632667	0.66	0.004
1E+08	LOC100045999	0.66	0.002
66448	Mrpl20	0.65	0.000
98238	Lrrc59	0.65	0.007
12825	Col3a1	0.65	0.036
52033	Pbk	0.65	0.010
67834	Idh3a	0.64	0.027
110172	Slc35b1	0.64	0.026
231440	9130213B05Rik	0.64	0.001
223917	Krt79	0.63	0.000
12314	Calm2	0.63	0.010
30058	Timm8a1	0.63	0.000
17993	Ndufs4	0.62	0.003
59029	Psmd14	0.62	0.001
13052	Cxadr	0.62	0.005
17448	Mdh2	0.62	0.005
12053	Bcl6	0.62	0.001
66092	Ghitm	0.62	0.001
60527	Fads3	0.61	0.018

11520	Adfp	0.60	0.003
636952	LOC636952	0.60	0.015
68614	Letmd1	0.59	0.026
66841	Etfdh	0.59	0.012
66961	2310043N10Rik	0.59	0.014
15490	Hsd17b7	0.59	0.021
227613	Tubb2c	0.59	0.049
67892	1810063B05Rik	0.58	0.001
53860	Sept9	0.57	0.007
66249	Pno1	0.57	0.001
639931	LOC639931	0.57	0.003
66925	Sdhd	0.57	0.003
215951	Lace1	0.57	0.001
78651	Lsm6	0.56	0.015
56438	Rbx1	0.56	0.003
217837	Itpk1	0.56	0.014
22027	Hsp90b1	0.56	0.001
677317	LOC677317	0.56	0.009
69702	Ndufaf1	0.56	0.000
68735	Mrps18c	0.56	0.008
12469	Cct8	0.55	0.000
11370	Acadvl	0.55	0.018
69064	1810014F10Rik	0.55	0.009
11370	Acadvl	0.55	0.050
16854	Lgals3	0.55	0.031
66218	Ndufb9	0.54	0.005
63953	Dusp10	0.54	0.000
15568	Elavl1	0.54	0.003
20194	S100a10	0.54	0.025
18102	Nme1	0.54	0.036
68016	2310039E09Rik	0.54	0.021
22628	Ywhag	0.53	0.010
667190	EG667190	0.53	0.017
66834	Them2	0.53	0.013
19285	Ptrf	0.53	0.005
71941	Cars2	0.53	0.001
17984	Ndn	0.53	0.046
269831	Tspan12	0.52	0.046
12387	Ctnnb1	0.52	0.009
14791	Emg1	0.52	0.019
231889	Bud31	0.52	0.002
14228	Fkbp4	0.52	0.012
68493	1110007M04Rik	0.52	0.033
71679	Atp5h	0.51	0.004
67851	1700021F05Rik	0.51	0.005

16419	Itgb5	0.51	0.034
68342	Ndufb10	0.51	0.013
225887	Ndufs8	0.50	0.013
230075	Ndufb6	0.50	0.015
68263	Pdhb	0.50	0.003
1E+08	LOC100042777	0.50	0.021
12406	Serpinh1	0.50	0.008
12866	Cox7a2	0.50	0.001
78653	Bola3	0.49	0.028
66477	Usmg5	0.49	0.000
110842	Etfa	0.49	0.020
68836	Mrpl52	0.49	0.043
66117	1110001J03Rik	0.49	0.006
109006	Ciapin1	0.49	0.029
72043	Sulf2	0.49	0.004
21454	Tcp1	0.48	0.025
59052	Mettl9	0.48	0.000
56348	Hsd17b12	0.48	0.014
74600	Mrpl47	0.48	0.003
94063	Mrpl16	0.48	0.005
11911	Atf4	0.48	0.021
26908	Eif2s3y	0.48	0.001
26458	Slc27a2	0.48	0.041
52469	Ccdc56	0.47	0.022
27279	Tnfrsf12a	0.47	0.023
14793	Cdca3	0.47	0.024
66310	Dpy30	0.47	0.018
76281	Tax1bp3	0.47	0.021
22631	Ywhaz	0.46	0.038
18645	Pfn2	0.46	0.029
16905	Lmna	0.46	0.008
18645	Pfn2	0.46	0.036
75597	Ndufa12l	0.46	0.012
68198	Ndufb2	0.46	0.000
22171	Tyms	0.46	0.012
1E+08	LOC100047184	0.46	0.000
20133	Rrm1	0.46	0.041
17149	Magoh	0.46	0.006
21379	Tbrg4	0.46	0.002
18674	Slc25a3	0.46	0.000
56367	Scoc	0.45	0.002
12334	Capn2	0.45	0.005
215210	Tmem120a	0.45	0.017
60530	Fignl1	0.45	0.030
22172	Tyms-ps	0.45	0.029

229709	Ahcyl1	0.45	0.010
67877	Nat5	0.44	0.001
329252	Lgr6	0.44	0.001
76863	Dcun1d5	0.44	0.001
67283	Slc25a19	0.44	0.043
13382	Dld	0.44	0.005
78920	Dlst	0.44	0.037
15384	Hnrnpab	0.44	0.021
56075	Pdss1	0.44	0.000
99138	Stard7	0.44	0.008
26446	Psmb3	0.44	0.009
268697	Ccnb1	0.43	0.012
70247	Psmd1	0.43	0.021
76281	Tax1bp3	0.43	0.044
66433	Chchd7	0.43	0.016
12334	Capn2	0.43	0.006
69536	Hemk1	0.43	0.015
97064	Wwtr1	0.43	0.005
21877	Tk1	0.43	0.037
13592	Ebf2	0.43	0.005
67877	Nat5	0.43	0.009
638892	LOC638892	0.43	0.014
13063	Cycs	0.43	0.001
76614	Immt	0.42	0.006
634015	LOC634015	0.42	0.043
70186	Fam162a	0.42	0.049
20335	Sec61g	0.42	0.013
66047	Mrpl54	0.42	0.005
665369	EG665369	0.42	0.017
380773	1810035L17Rik	0.42	0.015
102866	Pls3	0.42	0.016
244810	AW551984	0.42	0.049
19299	Abcd3	0.42	0.005
1E+08	LOC100041703	0.42	0.026
1E+08	LOC100042773	0.42	0.035
12166	Bmpr1a	0.42	0.001
66106	Smpx	0.41	0.018
11852	Rhob	0.41	0.030
54683	Prdx5	0.41	0.020
50927	Nasp	0.41	0.007
104303	Arl1	0.41	0.003
19302	Pex2	0.41	0.006
83961	Nrg4	0.41	0.020
74183	2310042D19Rik	0.41	0.045
69046	Isca1	0.41	0.046

64655	Mrps22	0.41	0.012
105559	Mbnl2	0.41	0.001
56456	Actl6a	0.41	0.015
69674	Mif4gd	0.41	0.028
12334	Capn2	0.41	0.003
70231	Gorasp2	0.40	0.016
81799	C1qtnf3	0.40	0.008
52250	Reep1	0.40	0.048
68194	Ndufb4	0.40	0.010
23837	Cfdp1	0.40	0.009
66242	Mrps16	0.40	0.011
21854	Timm17a	0.40	0.017
70160	Vps36	0.40	0.018
216767	Mrpl22	0.40	0.020
13527	Dtna	0.40	0.015
19345	Rab5c	0.40	0.019
19359	Rad23b	0.40	0.005
71883	Coq2	0.40	0.013
66043	Atp5d	0.40	0.031
67117	Dynlt3	0.40	0.032
69478	2300009A05Rik	0.40	0.024
170760	Acbd3	0.39	0.005
63953	Dusp10	0.39	0.004
16673	Krt36	0.39	0.001
17846	Commd1	0.39	0.015
11544	Adprh	0.39	0.019
76813	Armc6	0.39	0.004
170755	Sgk3	0.39	0.004
80888	Hspb8	0.39	0.017
69487	2310003L22Rik	0.39	0.004
67710	Polr2g	0.38	0.010
14792	Lpcat3	0.38	0.007
67308	Mrpl46	0.38	0.027
69538	Antxr1	0.38	0.011
69956	Ptcd3	0.38	0.009
12289	Cacna1d	0.38	0.013
29858	Pmm1	0.38	0.004
19027	Sypl	0.38	0.022
13664	Eif1a	0.38	0.001
13877	Erh	0.38	0.036
1E+08	LOC100044756	0.38	0.043
18226	Nup62	0.38	0.013
11306	Abcb7	0.38	0.010
54630	Prickle3	0.38	0.001
54351	Rai12	0.38	0.014

236792	Tmem32	0.38	0.002
66397	Sar1b	0.38	0.038
56433	Vps29	0.37	0.016
72416	Lrpprc	0.37	0.015
54216	Pcdh7	0.37	0.005
66121	Chchd1	0.37	0.034
11428	Aco1	0.37	0.014
20383	Sfrs3	0.37	0.005
381760	Ssbp1	0.37	0.033
29864	Rnf11	0.37	0.028
66375	Dhrs7	0.37	0.031
67270	D10Ertd322e	0.37	0.032
108115	Slco4a1	0.37	0.007
19072	Prep	0.37	0.006
1E+08	LOC100045542	0.37	0.019
94066	Mrpl36	0.37	0.035
67204	Eif2s2	0.37	0.033
53605	Nap1l1	0.37	0.049
1E+08	LOC100047167	0.37	0.024
69071	Tmem97	0.37	0.042
230596	Prpf38a	0.37	0.016
321022	Cdv3	0.37	0.012
27401	Skp2	0.37	0.039
72900	Ndufv2	0.37	0.005
17436	Mod1	0.36	0.014
12305	Ddr1	0.36	0.000
16952	Anxa1	0.36	0.001
19655	Rbmx	0.36	0.008
70984	4931406C07Rik	0.36	0.032
11539	Adora1	0.36	0.003
12450	Ccng1	0.36	0.017
17713	Grpel1	0.36	0.045
77407	Rab35	0.36	0.007
66291	1810030N24Rik	0.36	0.009
99730	Taf13	0.36	0.000
14548	Mrps33	0.35	0.027
675985	LOC675985	0.35	0.026
68032	Tmem85	0.35	0.011
69072	Ebna1bp2	0.35	0.002
74596	Cds1	0.35	0.013
68460	Dhrs7c	0.35	0.033
27407	Abcf2	0.35	0.010
229589	Prune	0.35	0.025
270163	Myo9a	0.35	0.011
76306	1110021L09Rik	0.35	0.009

224904	2410015M20Rik	0.35	0.020
1E+08	LOC100046343	0.35	0.043
434632	BC085271	0.35	0.035
104725	1110002B05Rik	0.34	0.039
20383	Sfrs3	0.34	0.004
66671	Ccnh	0.34	0.024
70503	Ddo	0.34	0.005
16905	Lmna	0.34	0.047
15507	Hspb1	0.34	0.026
74205	Acsl3	0.34	0.033
215751	BC013529	0.34	0.045
70231	Gorasp2	0.34	0.025
26754	Cops5	0.34	0.005
29876	Clic4	0.34	0.024
110651	Rps6ka3	0.33	0.009
210146	Irgq	0.33	0.014
386612	Thoc6	0.33	0.010
76178	6330578E17Rik	0.33	0.042
75991	Slain2	0.33	0.006
66048	Tmem93	0.33	0.014
70122	Mllt3	0.33	0.035
1E+08	LOC100041500	0.33	0.038
407785	Ndufs6	0.33	0.036
78248	Armcx1	0.33	0.020
1E+08	LOC100047794	0.33	0.004
26754	Cops5	0.33	0.026
68197	Ndufc2	0.33	0.005
108098	Med21	0.32	0.010
665509	EG665509	0.32	0.005
66849	Ppp1r2	0.32	0.032
626152	LOC626152	0.32	0.020
19247	Ptpn11	0.32	0.020
67994	Mrps11	0.32	0.031
237781	Smcr7	0.32	0.022
66587	Fastk	0.32	0.032
12387	Ctnnb1	0.32	0.036
64833	Acot10	0.32	0.011
231440	9130213B05Rik	0.32	0.030
66365	Ccdc90b	0.31	0.005
19183	Psmc3ip	0.31	0.020
52840	Dbndd2	0.31	0.034
235339	Dlat	0.31	0.020
118451	Mrps2	0.31	0.000
67894	1810055E12Rik	0.31	0.031
76485	Glt8d1	0.31	0.001

16646	Kpna1	0.31	0.001
26556	Homer1	0.31	0.020
12023	Barx2	0.31	0.026
18105	Nqo2	0.31	0.037
214952	Rhot2	0.31	0.041
56282	Mrpl12	0.31	0.026
74868	Tmem65	0.31	0.049
211347	Pank3	0.30	0.040
114641	Rpl31	0.30	0.022
16780	Lamb3	0.30	0.006
59009	Sh3rf1	0.30	0.023
28028	Mrpl50	0.30	0.044
66993	Smarcd3	0.30	0.036
97820	4833439L19Rik	0.30	0.040
98417	Cnih4	0.30	0.018
67863	Slc25a11	0.30	0.048
11832	Aqp7	0.30	0.048
107765	Ankrd1	0.30	0.001
50529	Mrps7	0.30	0.022
68523	1110019N10Rik	0.30	0.012
66665	5730528L13Rik	0.29	0.004
80752	BC004044	0.29	0.005
16600	Klf4	0.29	0.023
76551	Ccdc6	0.29	0.031
213491	D4Ertd22e	0.29	0.010
67921	Ube2f	0.29	0.043
272551	Gins2	0.29	0.026
68082	Dusp19	0.29	0.004
67609	4930453N24Rik	0.29	0.008
78521	B230219D22Rik	0.29	0.046
16579	Kifap3	0.29	0.007
68032	Tmem85	0.29	0.043
14007	Cugbp2	0.29	0.026
545388	B020018G12Rik	0.29	0.044
28030	Gfm1	0.29	0.014
213541	Ythdf2	0.29	0.018
14791	Emg1	0.29	0.046
54351	Rai12	0.29	0.005
56200	Ddx21	0.29	0.042
64934	Pes1	0.29	0.005
68724	Arl8a	0.28	0.029
26572	Cops3	0.28	0.046
69882	2010321M09Rik	0.28	0.011
1E+08	LOC100043675	0.28	0.032
79560	Ublcp1	0.28	0.027

74006	Dnm1l	0.28	0.046
67888	Tmem100	0.28	0.026
22200	Ube1c	0.28	0.015
66997	Psmd12	0.28	0.032
11677	Akr1b3	0.28	0.013
21402	Skp1a	0.27	0.046
72175	Mfsd8	0.27	0.015
66410	Mterfd1	0.27	0.007
74006	Dnm1l	0.27	0.031
64656	Mrps23	0.27	0.023
104771	1200003C05Rik	0.27	0.016
71934	Car13	0.27	0.049
108099	Prkag2	0.27	0.050
269424	Phf17	0.27	0.038
114663	Impa2	0.27	0.022
108124	Napa	0.27	0.001
69861	2010003K11Rik	0.27	0.036
21906	Otop1	0.27	0.010
70356	St13	0.27	0.014
110750	Cse1l	0.26	0.008
213436	Zcchc5	0.26	0.012
99045	Mrps26	0.26	0.015
16211	Kpnb1	0.26	0.021
80752	BC004044	0.26	0.000
71913	Tmem79	0.26	0.004
60411	Cenpk	0.26	0.024
67894	1810055E12Rik	0.26	0.031
230709	Zmpste24	0.26	0.014
97440	B3gnt9	0.26	0.005
70257	2010107E04Rik	0.26	0.025
65112	Pmepa1	0.26	0.001
59046	Arpp19	0.26	0.032
101437	Dhx32	0.26	0.023
217737	Ahsa1	0.26	0.047
93696	Chrac1	0.26	0.014
69136	Tusc1	0.26	0.014
234865	Nup133	0.26	0.010
69639	Exosc8	0.26	0.025
15159	Hccs	0.25	0.004
329910	Acot11	0.25	0.037
17919	Myo5b	0.25	0.043
12144	Blm	0.25	0.015
57896	Krcc1	0.25	0.036
243813	Leng9	0.25	0.013
72421	Ttc30b	0.25	0.021

11769	Ap1s1	0.25	0.033
74838	Narg1	0.25	0.014
214572	Prmt7	0.25	0.016
229363	Gmps	0.25	0.016
59048	C1galt1c1	0.25	0.041
29819	Stau2	0.25	0.001
12830	Col4a5	0.25	0.039
74646	Spsb1	0.25	0.014
70885	Ints10	0.25	0.049
26992	Brd7	0.25	0.008
227743	Mapkap1	0.25	0.025
67204	Eif2s2	0.25	0.041
74132	Rnf6	0.25	0.046
98238	Lrrc59	0.25	0.004
14356	Fxc1	0.24	0.045
12181	Bop1	0.24	0.005
233876	Hirip3	0.24	0.045
72692	Hnrpll	0.24	0.029
22248	Unc119	0.24	0.013
11518	Add1	0.24	0.024
386649	Nsfl1c	0.24	0.049
23885	Gmcl1	0.24	0.044
56280	Mrpl37	0.24	0.032
105446	Gmpr2	0.24	0.030
18008	Nes	0.24	0.041
109093	Rars2	0.24	0.041
68291	Mto1	0.24	0.006
27377	Yme1l1	0.24	0.008
83997	Slmap	0.23	0.043
235599	6430571L13Rik	0.23	0.014
56748	Nfu1	0.23	0.014
66821	Bcs1l	0.23	0.036
207952	Klhl25	0.23	0.008
14745	Edg2	0.23	0.019
20016	Polr1c	0.23	0.016
69077	Psmd11	0.23	0.012
545472	LOC545472	0.23	0.011
244962	Snx14	0.23	0.022
109168	5730596K20Rik	0.23	0.013
14376	Ganab	0.23	0.043
67046	Tbc1d7	0.23	0.040
12450	Ccng1	0.23	0.024
1E+08	LOC100044842	0.23	0.018
230654	Lrrc41	0.23	0.027
66398	Commd5	0.23	0.043

1E+08	LOC100047911	0.23	0.024
56150	Mad2l1	0.22	0.048
208628	Kntc1	0.22	0.037
52575	Rg9mtd1	0.22	0.014
109168	Atl3	0.22	0.005
22019	Tpp2	0.22	0.011
66595	Aste1	0.22	0.029
16673	Krt36	0.22	0.002
14356	Fxc1	0.22	0.045
66902	Mtap	0.22	0.013
104458	Rars	0.22	0.023
68092	Ncbp2	0.22	0.033
65111	Dap3	0.22	0.041
70153	2210016F16Rik	0.22	0.036
22171	Tyms	0.22	0.025
56279	B230317C12Rik	0.22	0.003
13167	Dbi	0.22	0.037
66870	Serbp1	0.22	0.034
224020	Pi4ka	0.22	0.043
66067	Gtpbp8	0.21	0.004
56279	B230317C12Rik	0.21	0.019
52715	Ccdc43	0.21	0.024
74467	Pus10	0.21	0.018
11982	Atp10a	0.21	0.012
76983	Scfd1	0.21	0.018
225745	Ccdc5	0.21	0.030
72124	Seh1l	0.21	0.012
50786	Hs6st2	0.21	0.037
237880	1700071K01Rik	0.21	0.013
66523	2810004N23Rik	0.21	0.017
101631	Pwwp2b	0.20	0.049
70673	Prdm16	0.20	0.004
17975	Ncl	0.20	0.046
77116	Mtmr2	0.20	0.041
26374	Rfwd2	0.20	0.015
99890	Prmt6	0.20	0.042
66912	Bzw2	0.20	0.049
66997	Psmd12	0.20	0.025
66314	Tpd52l2	0.20	0.016
1E+08	LOC100039175	0.20	0.022
20411	Sorbs1	0.20	0.041
68184	Denr	0.20	0.030
18693	Pick1	0.20	0.031
20972	Syngr1	0.20	0.029
26556	Homer1	0.20	0.038

19376	Rab34	0.20	0.013
12832	Col5a2	0.20	0.018
105440	Kctd9	0.20	0.046
76167	6330548G22Rik	0.19	0.013
67456	Ergic2	0.19	0.035
99650	4933434E20Rik	0.19	0.015
338359	Supv3l1	0.19	0.025
70394	Kptn	0.19	0.020
66136	Znrd1	0.19	0.040
99890	Prmt6	0.19	0.040
21353	Tank	0.19	0.030
12579	Cdkn2b	0.19	0.031
77116	Mtmr2	0.19	0.024
66165	Bccip	0.19	0.045
245841	Polr2h	0.19	0.048
68537	Mrpl13	0.19	0.043
106931	Kctd1	0.19	0.009
21406	Tcf12	0.19	0.015
71703	Armcx3	0.19	0.045
76273	Ndfip2	0.18	0.031
23885	Gmcl1	0.18	0.046
13690	Eif4g2	0.18	0.022
71988	Esco2	0.18	0.032
228491	Zfp770	0.18	0.024
12873	Cpa3	0.18	0.022
56749	Dhodh	0.18	0.045
629763	LOC629763	0.18	0.025
218441	Zfyve16	0.18	0.042
67014	Mina	0.18	0.020
66497	2610528E23Rik	0.17	0.023
77781	Epm2aip1	0.17	0.017
71690	Esm1	0.17	0.023
16562	Kif1c	0.17	0.032
19336	Rab24	0.17	0.025
73274	Gpbp1	0.17	0.042
94254	Wbscr16	0.17	0.046
72354	Ttc4	0.17	0.047
11910	Atf3	0.16	0.041
50915	Grb14	0.16	0.048
66185	1110037F02Rik	0.16	0.032
27395	Mrpl15	0.15	0.027
14231	Fkbp7	0.15	0.050
234736	Rfwd3	0.15	0.044
236904	Klhl15	0.15	0.045
67391	Fundc2	0.15	0.022

70240	Ufsp1	0.15	0.032
1E+08	LOC100047052	0.15	0.034
12837	Col8a1	0.15	0.005
18648	Pgam1	0.15	0.045
67477	1300007F04Rik	0.15	0.005
229731	Slc25a24	0.15	0.036
320365	Fry	0.14	0.023
240892	Dusp27	0.14	0.011
12043	Bcl2	0.14	0.037
71711	Mus81	0.14	0.020
22689	Zfp27	0.14	0.042
109245	Lrrc39	0.14	0.003
72139	2610044O15Rik	0.14	0.040
225579	Slc27a6	0.13	0.022
73016	Kremen2	0.13	0.032
75424	Zfp820	0.13	0.022
103765	Tmem17	0.13	0.008
69608	Sec24d	0.13	0.040
69944	2810021J22Rik	0.13	0.030
70788	Klhl30	0.13	0.032
22688	Zfp26	0.12	0.020
22337	Vdr	0.12	0.022
76854	Gper	0.12	0.028
68760	Synpo2l	0.12	0.049
1E+08	LOC100045958	0.12	0.048
384596	EG384596	0.12	0.049
71151	Exod1	0.12	0.048
1E+08	LOC100041194	0.12	0.019
63993	Slc5a7	0.11	0.043
320234	Ccdc66	0.11	0.044
56369	Apip	0.10	0.023
68857	Dtwd2	0.10	0.042
71521	Pds5a	0.10	0.029
19124	Procr	0.10	0.047
20378	Frzb	0.09	0.041
68775	Atp6v1c2	0.07	0.044
319748	6430526N21Rik	-0.09	0.027
239559	A4galt	-0.09	0.048
77533	C030034I22Rik	-0.09	0.046
57780	Fxyd7	-0.10	0.049
239647	Fam113b	-0.10	0.029
72296	Rusc1	-0.11	0.049
75561	1700016J18Rik	-0.12	0.002
14128	Fcer2a	-0.12	0.043
171580	Mical1	-0.12	0.038

214897	Csnk1g1	-0.13	0.042
104418	Dgkz	-0.13	0.038
72140	Ccdc123	-0.13	0.036
214547	She	-0.13	0.029
103724	Tbc1d10a	-0.14	0.044
30843	Fbxl12	-0.14	0.043
676136	LOC676136	-0.14	0.048
1E+08	LOC100048504	-0.15	0.045
320712	Abi3bp	-0.15	0.011
18720	Pip5k1a	-0.15	0.027
19775	Xpr1	-0.15	0.043
215705	Arrdc1	-0.15	0.034
71148	Mier1	-0.15	0.039
116848	Baz2a	-0.16	0.047
140484	Pofut1	-0.16	0.042
319713	Ablim3	-0.16	0.027
18541	Pcnt	-0.16	0.036
20555	Slfn1	-0.16	0.024
27886	Dgcr14	-0.16	0.018
245865	Spag4	-0.17	0.011
12702	Socs3	-0.17	0.046
30838	Fbxw4	-0.17	0.032
94094	Trim34	-0.17	0.036
20947	Swap70	-0.17	0.018
67661	Ift172	-0.17	0.031
14182	Fgfr1	-0.18	0.005
245847	Amdhd2	-0.18	0.009
243612	D630042P16Rik	-0.18	0.032
68895	Rasl11a	-0.18	0.012
52609	Cbx7	-0.18	0.030
69434	Snhg10	-0.18	0.007
71923	2310047M10Rik	-0.18	0.038
229003	BC006779	-0.18	0.031
103677	Smg6	-0.18	0.032
67200	Ccdc77	-0.18	0.033
77110	Gpbp1l1	-0.19	0.012
15184	Hdac5	-0.19	0.028
56321	Aatf	-0.19	0.036
57354	Cramp1l	-0.19	0.017
403202	A430093F15Rik	-0.19	0.016
217119	Xylt2	-0.19	0.016
76707	Clasp1	-0.19	0.023
67769	Gpatch2	-0.19	0.045
28081	D11Wsu99e	-0.19	0.023
78816	Gmip	-0.19	0.029

1E+08	LOC100044439	-0.19	0.018
12493	Cd37	-0.19	0.040
20181	Rxra	-0.19	0.048
101706	Numa1	-0.19	0.030
76187	Adhfe1	-0.19	0.022
107986	Ddb2	-0.19	0.047
1E+08	LOC100043189	-0.19	0.039
20840	Stac	-0.20	0.027
1E+08	LOC100040462	-0.20	0.047
57276	Vsig2	-0.20	0.021
232201	Arhgap25	-0.20	0.011
21407	Tcf15	-0.20	0.008
75234	Ibrdc3	-0.20	0.005
17912	Myo1b	-0.20	0.047
55943	Stx8	-0.20	0.033
100756	Usp30	-0.20	0.043
98999	Znfx1	-0.20	0.026
664862	Gpr137b-ps	-0.20	0.038
57783	Tnip1	-0.20	0.015
78610	Uvrag	-0.20	0.012
214968	Sema6d	-0.20	0.042
16970	Lrmp	-0.20	0.015
23856	Dido1	-0.20	0.046
216505	Pik3ip1	-0.20	0.018
76051	Ganc	-0.21	0.009
68947	Chst8	-0.21	0.012
11432	Acp2	-0.21	0.029
16188	Il3ra	-0.21	0.034
1E+08	LOC100044324	-0.21	0.036
21400	Tcea2	-0.21	0.012
277414	Trp53i11	-0.21	0.006
76187	Adhfe1	-0.21	0.004
330474	BC057627	-0.21	0.018
56743	Lat2	-0.21	0.043
225888	Suv420h1	-0.21	0.031
68501	Nsmce2	-0.21	0.022
75410	Wbp7	-0.21	0.032
56464	Ctsf	-0.21	0.045
434484	Sp140	-0.21	0.001
215193	AA408296	-0.21	0.012
216869	Arrb2	-0.22	0.009
20403	Itsn2	-0.22	0.015
72823	Pard3b	-0.22	0.004
210710	Gab3	-0.22	0.027
72948	Tppp	-0.22	0.040

74195	Elp3	-0.22	0.028
69654	Dctn2	-0.22	0.043
73447	Wdr13	-0.22	0.043
12752	Cln3	-0.22	0.004
78816	Gmip	-0.22	0.005
224624	Rab40c	-0.22	0.034
12748	Clk2	-0.22	0.013
13831	Epc1	-0.22	0.015
246177	Myo1g	-0.22	0.038
58230	Rnf8	-0.22	0.019
236920	Stard8	-0.23	0.016
66840	Wdr45l	-0.23	0.030
71279	Slc29a3	-0.23	0.010
105841	Dennd3	-0.23	0.016
17095	Lyl1	-0.23	0.027
223642	Zc3h3	-0.23	0.008
67145	Tomm34	-0.23	0.009
98685	1190005F20Rik	-0.23	0.049
19165	Psen2	-0.23	0.013
78308	Gpr108	-0.23	0.043
66505	Zmynd11	-0.23	0.039
98732	Rab3gap2	-0.23	0.020
78334	Cdc2l6	-0.23	0.027
14708	Gng7	-0.23	0.016
107375	Slc25a45	-0.23	0.023
105352	Dusp22	-0.23	0.032
76051	Ganc	-0.23	0.005
20928	Abcc9	-0.23	0.014
17344	Pias2	-0.23	0.041
231866	Zfp12	-0.23	0.007
212483	BC021381	-0.23	0.039
83429	Ctns	-0.23	0.001
75731	5133401N09Rik	-0.23	0.021
68020	2810002N01Rik	-0.24	0.036
68731	1110032A13Rik	-0.24	0.028
245866	Ift52	-0.24	0.026
59024	Med12	-0.24	0.043
71175	Nipbl	-0.24	0.025
11819	Nr2f2	-0.24	0.005
20544	Slc9a1	-0.24	0.029
330474	BC057627	-0.24	0.041
19062	Inpp5k	-0.24	0.009
54402	Stk19	-0.24	0.013
215653	Rassf2	-0.24	0.031
56458	Foxo1	-0.24	0.027

20848	Stat3	-0.24	0.045
110147	Ehmt2	-0.24	0.040
232078	Thnsl2	-0.24	0.029
1E+08	LOC100048299	-0.24	0.011
13353	Dgcr6	-0.25	0.005
212398	Frat2	-0.25	0.008
245866	Ift52	-0.25	0.042
68552	1110003E01Rik	-0.25	0.040
108735	Sft2d2	-0.25	0.006
381629	0610007C21Rik	-0.25	0.043
108655	Foxp1	-0.25	0.010
16197	Il7r	-0.25	0.034
67488	Calcoco1	-0.25	0.018
20672	Sox18	-0.26	0.007
67186	Rplp2	-0.26	0.024
235504	Slc17a5	-0.26	0.040
69903	Rasip1	-0.26	0.014
140721	Caskin2	-0.26	0.005
224109	Lrrc33	-0.26	0.027
12554	Cdh13	-0.26	0.001
209200	Dtx3l	-0.26	0.016
66530	Ubxn6	-0.26	0.043
17874	Myd88	-0.26	0.027
69723	Rpain	-0.26	0.011
80911	Acox3	-0.26	0.005
21354	Tap1	-0.26	0.036
319565	Syne2	-0.27	0.011
98365	Slamf9	-0.27	0.023
22379	Fmnl3	-0.27	0.041
233833	Tnrc6a	-0.27	0.021
18131	Notch3	-0.27	0.022
71732	Vps11	-0.27	0.002
434204	Whdc1	-0.27	0.029
105827	Amigo2	-0.27	0.029
69274	Ctdspl	-0.27	0.010
24044	Scamp2	-0.27	0.015
233806	Tmem159	-0.28	0.002
74761	Mxra8	-0.28	0.004
56407	Trpc4ap	-0.28	0.029
15478	Hs3st3a1	-0.28	0.035
17939	Naga	-0.28	0.029
67880	Dcxr	-0.28	0.038
105844	Card10	-0.28	0.014
628101	LOC628101	-0.28	0.014
27416	Abcc5	-0.28	0.013

14387	Gaa	-0.28	0.018
110198	Akr7a5	-0.28	0.025
101602	AI467606	-0.28	0.039
22637	Zap70	-0.28	0.030
66990	Tmem134	-0.28	0.013
15018	H2-Q7	-0.28	0.048
68126	Fahd2a	-0.28	0.040
66404	2410001C21Rik	-0.28	0.032
15239	Hgs	-0.28	0.036
16994	Ltb	-0.29	0.016
1E+08	LOC100045877	-0.29	0.034
77590	4631426J05Rik	-0.29	0.008
70575	Gfod2	-0.29	0.001
17346	Mknk1	-0.29	0.032
18417	Cldn11	-0.29	0.018
213573	Efcab4a	-0.29	0.011
69745	Pold4	-0.29	0.026
76233	Dnttip1	-0.29	0.037
107723	Slc12a6	-0.29	0.026
17207	Mcf2l	-0.29	0.032
214597	Sidt2	-0.29	0.011
70785	Dennd1c	-0.29	0.007
71361	Aifm2	-0.29	0.009
12125	Bcl2l11	-0.29	0.018
17207	Mcf2l	-0.29	0.010
54215	Cd160	-0.29	0.032
675228	LOC675228	-0.29	0.014
18613	Pecam1	-0.29	0.005
68792	Srpx2	-0.29	0.024
243374	Gimap8	-0.29	0.003
110094	Phka2	-0.29	0.019
78887	Sfi1	-0.29	0.049
229542	Gatad2b	-0.29	0.002
16428	Itk	-0.30	0.008
104110	Adcy4	-0.30	0.025
55934	rp9	-0.30	0.044
18132	Notch4	-0.30	0.013
20130	Rras	-0.30	0.037
54208	Arl6ip1	-0.30	0.024
66566	2310079N02Rik	-0.30	0.018
72282	1810062G17Rik	-0.30	0.005
218963	LOC218963	-0.30	0.047
269608	Plekhg5	-0.30	0.001
233016	Blvrb	-0.30	0.041
66625	5730406M06Rik	-0.30	0.012

71956	Rnf135	-0.30	0.005
214597	Sidt2	-0.31	0.018
108687	Edem2	-0.31	0.024
56030	Tmem131	-0.31	0.030
108155	Ogt	-0.31	0.013
19128	Pros1	-0.31	0.019
67905	Ppm1m	-0.31	0.026
20356	Sema5a	-0.32	0.035
14612	Gja4	-0.32	0.004
20661	Sort1	-0.32	0.001
67188	2700046G09Rik	-0.32	0.025
20315	Cxcl12	-0.32	0.017
97287	Mtmr14	-0.32	0.030
102084	AI451557	-0.32	0.003
110147	Ehmt2	-0.33	0.008
14247	Fli1	-0.33	0.003
16432	Itm2b	-0.33	0.044
108155	Ogt	-0.33	0.017
11797	Birc2	-0.33	0.021
16625	Serpina3c	-0.33	0.042
23789	Coro1b	-0.33	0.031
15896	Icam2	-0.33	0.029
108705	Pttg1ip	-0.33	0.014
11972	Atp6v0d1	-0.33	0.018
104318	Csnk1d	-0.34	0.032
26406	Map3k3	-0.34	0.000
22187	Ubb	-0.34	0.035
207728	Pde2a	-0.34	0.000
18301	Fxyd5	-0.34	0.048
16541	Napsa	-0.34	0.005
54445	Unc93b1	-0.34	0.014
12757	Clta	-0.34	0.015
77006	Ddrgk1	-0.34	0.025
68592	Syf2	-0.34	0.022
16779	Lamb2	-0.34	0.008
227700	Sh3glb2	-0.34	0.012
99683	Sec24b	-0.34	0.001
1E+08	LOC100047093	-0.34	0.018
18010	Neu1	-0.35	0.039
110095	Pygl	-0.35	0.041
26429	Orc5l	-0.35	0.036
66366	Ergic3	-0.36	0.029
22378	Wbp2	-0.36	0.005
71706	Slc46a3	-0.36	0.007
15979	Ifngr1	-0.36	0.038

11826	Aqp1	-0.36	0.009
224139	Golgb1	-0.36	0.002
57342	Parva	-0.36	0.002
22324	Vav1	-0.37	0.014
11490	Adam15	-0.37	0.000
17159	Man2b1	-0.37	0.029
1E+08	LOC100048434	-0.37	0.000
16421	Itgb7	-0.37	0.028
217069	Trim25	-0.37	0.043
77006	2600009E05Rik	-0.37	0.010
19934	Rpl22	-0.37	0.038
13041	Ctsw	-0.37	0.015
83486	Rbm5	-0.37	0.040
12351	Car4	-0.37	0.044
19156	Psap	-0.38	0.032
320405	Cadps2	-0.38	0.024
624036	Gm1821	-0.38	0.028
246696	Slc25a28	-0.38	0.028
11450	Adipoq	-0.38	0.014
77087	Ankrd11	-0.38	0.029
268566	Gphn	-0.38	0.043
239743	Klhl6	-0.38	0.024
104318	Csnk1d	-0.38	0.033
14132	Fcgrt	-0.38	0.023
14705	Bscl2	-0.39	0.008
65962	Slc9a3r2	-0.39	0.017
1E+08	LOC100044190	-0.39	0.032
18220	Nucb1	-0.39	0.001
14470	Rabac1	-0.40	0.010
21940	Cd27	-0.40	0.047
105450	Mmrn2	-0.40	0.000
1E+08	LOC100044204	-0.40	0.030
13424	Dync1h1	-0.40	0.050
22117	Tst	-0.41	0.042
74030	Rin2	-0.41	0.014
57783	Tnip1	-0.41	0.026
54354	Rassf5	-0.41	0.021
26390	Mapkbp1	-0.41	0.022
50794	Klf13	-0.41	0.032
67880	Dcxr	-0.41	0.016
14057	Sfxn1	-0.41	0.036
114886	Cygb	-0.41	0.014
67168	P2ry5	-0.42	0.033
19414	Rasa3	-0.42	0.013
52430	Echdc2	-0.42	0.027

12039	Bckdha	-0.42	0.048
239743	Klhl6	-0.42	0.027
13350	Dgat1	-0.42	0.028
26936	Mprip	-0.43	0.011
67895	Ppa1	-0.43	0.003
52468	Ctdsp2	-0.43	0.009
69169	Faim3	-0.43	0.035
18574	Pde1b	-0.43	0.004
14824	Grn	-0.43	0.001
11826	Aqp1	-0.43	0.003
353156	Egfl7	-0.44	0.007
67784	Plxnd1	-0.44	0.007
76051	Ganc	-0.44	0.001
11722	Amy1	-0.44	0.031
67231	Tbc1d20	-0.44	0.008
13139	Dgka	-0.44	0.003
231712	Trafd1	-0.44	0.020
26943	Serinc3	-0.44	0.018
56312	Nupr1	-0.44	0.005
19301	Pxmp2	-0.45	0.027
20185	Ncor1	-0.45	0.016
231931	Gimap6	-0.45	0.004
106952	Centd3	-0.45	0.007
20621	Snn	-0.45	0.004
1E+08	LOC100048445	-0.45	0.016
214162	Mll1	-0.46	0.030
64817	Svep1	-0.46	0.000
20848	Stat3	-0.46	0.007
71712	1200002N14Rik	-0.47	0.002
12010	B2m	-0.47	0.047
66599	Rdm1	-0.47	0.003
84094	Plvap	-0.47	0.001
15211	Hexa	-0.47	0.011
22371	Vwf	-0.47	0.012
14874	Gstz1	-0.48	0.027
1E+08	LOC100047214	-0.48	0.024
80880	Ankrd47	-0.48	0.005
11669	Aldh2	-0.48	0.005
11752	Anxa8	-0.48	0.007
13716	Ell	-0.48	0.033
76969	Chst1	-0.48	0.002
26938	St6galnac5	-0.48	0.008
66612	Ormdl3	-0.48	0.040
77866	E130102H24Rik	-0.49	0.047
269275	Acvr1c	-0.49	0.009

56738	Mocs1	-0.49	0.045
18035	Nfkbia	-0.50	0.047
17200	Mc2r	-0.50	0.000
16205	Gimap1	-0.50	0.001
74392	Specc1l	-0.50	0.014
76263	Gstk1	-0.51	0.006
17681	Msc	-0.51	0.004
54194	Akap8l	-0.51	0.001
224792	Gpr116	-0.51	0.001
319899	Dock6	-0.51	0.002
12263	C2	-0.51	0.018
140742	Sesn1	-0.51	0.029
22695	Zfp36	-0.51	0.024
52685	Cd300lg	-0.52	0.010
70719	Hmha1	-0.52	0.016
1E+08	LOC100047126	-0.52	0.004
12010	B2m	-0.53	0.027
20230	Satb1	-0.53	0.019
12562	Cdh5	-0.53	0.000
68453	Gpihbp1	-0.54	0.002
14667	Gm2a	-0.54	0.001
78754	Galntl2	-0.55	0.015
1E+08	LOC100044204	-0.56	0.021
18613	Pecam1	-0.56	0.000
19062	Inpp5k	-0.56	0.002
12496	Entpd2	-0.56	0.000
11537	Cfd	-0.56	0.027
12036	Bcat2	-0.57	0.005
233103	4931406P16Rik	-0.57	0.013
16000	Igf1	-0.58	0.044
74144	Robo4	-0.58	0.009
394430	Ugt1a10	-0.59	0.016
65963	Tmem176b	-0.59	0.029
56357	Ivd	-0.59	0.000
13139	Dgka	-0.59	0.009
76491	Abhd14b	-0.60	0.000
101540	Prkd2	-0.60	0.000
26464	Vnn3	-0.60	0.003
18708	Pik3r1	-0.60	0.031
80885	Gpr109a	-0.61	0.007
14204	Il4i1	-0.61	0.013
110095	Pygl	-0.61	0.002
66058	Tmem176a	-0.61	0.001
56078	Car5b	-0.61	0.038
108995	Tbc1d10c	-0.62	0.014

246747	BC054059	-0.62	0.000
12192	Zfp36l1	-0.62	0.006
26464	Vnn3	-0.62	0.004
100637	B230342M21Rik	-0.62	0.039
227753	Gsn	-0.63	0.006
23972	Papss2	-0.63	0.023
394430	Ugt1a10	-0.64	0.005
83965	Enpp5	-0.64	0.001
66990	Tmem134	-0.65	0.000
235320	Zbtb16	-0.65	0.040
15040	H2-T23	-0.65	0.030
12226	Btg1	-0.65	0.049
16010	Igfbp4	-0.66	0.000
101488	Slco2b1	-0.69	0.010
230857	Ece1	-0.70	0.000
214597	Sidt2	-0.70	0.001
22041	Trf	-0.71	0.011
23871	Ets1	-0.71	0.000
11522	Adh1	-0.71	0.009
56349	Net1	-0.73	0.049
235493	BC031353	-0.74	0.020
227929	Cytip	-0.75	0.013
641240	LOC641240	-0.76	0.036
14775	Gpx1	-0.78	0.008
625018	C4a	-0.78	0.040
12741	Cldn5	-0.78	0.000
20363	Sepp1	-0.80	0.014
15439	Hp	-0.81	0.030
66168	Grina	-0.82	0.001
1E+08	LOC100046120	-0.86	0.024
108682	Gpt2	-0.86	0.001
234564	AU018778	-0.87	0.000
12526	Cd8b1	-0.88	0.028
14964	H2-D1	-0.90	0.011
66853	Pnpla2	-0.90	0.007
19122	Prnp	-0.91	0.001
1E+08	LOC100045864	-0.94	0.012
16149	Cd74	-0.94	0.025
12268	C4b	-0.94	0.008
674135	LOC674135	-0.97	0.007
66090	Ypel3	-0.97	0.004
625018	C4a	-0.98	0.004
1E+08	LOC100047628	-0.99	0.045
16149	Cd74	-0.99	0.034
20512	Slc1a3	-1.01	0.001

630499	EG630499	-1.01	0.005
54613	St3gal6	-1.09	0.008
56338	Txnip	-1.13	0.007
56338	Txnip	-1.24	0.004
20887	Sult1a1	-1.29	0.002
109959	Amy2-2	-1.48	0.001
665506	LOC665506	-1.50	0.029
104158	Ces3	-1.54	0.000
