# Supplementary material for: Prenatal Metformin Exposure in a Maternal High Fat Diet Mouse Model Alters the Transcriptome and Modifies the Metabolic Responses of the Offspring
Source: PLoS One. 2014 Dec 26;9(12):e115778. doi: 10.1371/journal.pone.0115778 (PMC4277397; doi:10.1371/journal.pone.0115778)
Supplement: S6 Table — Enriched pathways in the liver and SAT according to the hypergeometric test. q-value equals to P-value. (PDF) [file pone.0115778.s008.pdf]

Table S6. Enriched pathways in the liver and SAT according to the hypergeometric test.

q-value equals to P-value.

	Pathway REACTOME: SAT	q-value
1	The citric acid (TCA) cycle and respiratory electron transport	0.000
2	Respiratory electron transport, ATP synthesis by chemiosmotic coupling, and heat production by uncoupling proteins	0.000
3	Citric acid cycle (TCA cycle)	0.000
4	Pyruvate metabolism and Citric Acid (TCA) cycle	0.000
5	Respiratory electron transport	0.000
6	Antigen Presentation: Folding, assembly and peptide loading of class I MHC	0.001
7	Apoptotic execution phase	0.001
8	Class I MHC mediated antigen processing & presentation	0.001
9	Regulation of mRNA Stability by Proteins that Bind AU-rich Elements	0.004
10	Centrosome maturation	0.006
11	G2/M Transition	0.006
12	Recruitment of mitotic centrosome proteins and complexes	0.006
13	Mitotic G2-G2/M phases	0.008
14	Regulation of Cholesterol Biosynthesis by SREBP (SREBF)	0.008
15	Antigen processing-Cross presentation	0.009
16	ER-Phagosome pathway	0.009
17	Apoptosis	0.010
18	Muscle contraction	0.012
19	RNA Polymerase I Transcription Initiation	0.012
20	Striated Muscle Contraction	0.012
21	Nuclear signaling by ERBB4	0.018
22	Gluconeogenesis	0.023
23	Signaling by NOTCH	0.025
24	Destabilization of mRNA by AUF1 (hnRNP D0)	0.030
25	Loss of Nlp from mitotic centrosomes	0.030
26	Loss of proteins required for interphase microtubule organization from the centrosome	0.030
27	Cyclin A:Cdk2-associated events at S phase entry	0.032
28	Cyclin E associated events during G1/S transition	0.032
29	SCF(Skp2)-mediated degradation of p27/p21	0.033
30	Apoptotic cleavage of cellular proteins	0.034
31	Cooperation of Prefoldin and TriC/CCT in actin and tubulin folding	0.038
32	Degradation of beta-catenin by the destruction complex	0.038
33	Glucose metabolism	0.038
34	Immunoregulatory interactions between a Lymphoid and a non-Lymphoid cell	0.038
35	Prefoldin mediated transfer of substrate to CCT/TriC	0.038
36	Signaling by Wnt	0.038

37	APC/C-mediated degradation of cell cycle proteins	0.042
38	Regulation of mitotic cell cycle	0.042
39	Autodegradation of the E3 ubiquitin ligase COP1	0.043
40	Chaperonin-mediated protein folding	0.043
41	Cyclin D associated events in G1	0.043
42	G1 Phase	0.043
43	G1/S Transition	0.049
44	Stabilization of p53	0.050
	Pathway KEGG: SAT	q-value
1	Citrate cycle (TCA cycle)	0.000
2	Fatty acid metabolism	0.004
3	PPAR signaling pathway	0.004
4	Pyruvate metabolism	0.004
5	Valine, leucine and isoleucine degradation	0.004
6	Glycolysis / Gluconeogenesis	0.006
7	Small cell lung cancer	0.010
8	Starch and sucrose metabolism	0.019
9	Adipocytokine signaling pathway	0.033
10	Cysteine and methionine metabolism	0.033
11	Phosphatidylinositol signaling system	0.035
	Pathway REACTOME: Liver	q-value
1	Cytochrome P450 - arranged by substrate type	0.000
2	Phase 1 - Functionalization of compounds	0.001
3	Respiratory electron transport, ATP synthesis by chemiosmotic coupling, and heat production by uncoupling proteins	0.002
4	The citric acid (TCA) cycle and respiratory electron transport	0.002
	Pathway KEGG: Liver	q-value
1	Drug metabolism - other enzymes	0.000
2	Chemical carcinogenesis	0.000
3	Drug metabolism - cytochrome P450	0.000
4	Metabolism of xenobiotics by cytochrome P450	0.000
5	Valine, leucine and isoleucine degradation	0.000
6	Complement and coagulation cascades	0.000
7	Retinol metabolism	0.000
