# Supplementary material for: Prenatal Metformin Exposure in a Maternal High Fat Diet Mouse Model Alters the Transcriptome and Modifies the Metabolic Responses of the Offspring
Source: PLoS One. 2014 Dec 26;9(12):e115778. doi: 10.1371/journal.pone.0115778 (PMC4277397; doi:10.1371/journal.pone.0115778)
Supplement: S7 Table — Genes associated to REACTOME pathway citric acid (TCA) cycle and respiratory electron transport (ETC). (PDF) [file pone.0115778.s009.pdf]

Table S7. Genes associated to REACTOME pathway citric acid (TCA) cycle and respiratory electron transport (ETC).

The citric acid (TCA) cycle and respiratory electron transport			
Entrez	Symbol	Description	FoldChange
Liver			
11949	Atp5c1	ATP synthase, H+ transporting, mitochondrial F1 complex, gamma polypeptide 1	-0.393
14194	Fh1	fumarate hydratase 1	-0.688
17993	Ndufs4	NADH dehydrogenase (ubiquinone) Fe-S protein 4	-0.549
27425	Atp5l	ATP synthase, H+ transporting, mitochondrial F0 complex, subunit g	-0.481
66576	Uqcrh	ubiquinol-cytochrome c reductase hinge protein	-0.457
66841	Etfdh	electron transferring flavoprotein, dehydrogenase	-0.548
SAT			
110842	Etfa	electron transferring flavoprotein, alpha polypeptide	0.58
11946	Atp5a1	ATP synthase, H+ transporting, mitochondrial F1 complex, alpha subunit 1	1.054
13063	Cycs	cytochrome c, somatic	0.598
13382	Dld	dihydrolipoamide dehydrogenase	0.44
17448	Mdh2	malate dehydrogenase 2, NAD (mitochondrial)	0.624
17993	Ndufs4	NADH dehydrogenase (ubiquinone) Fe-S protein 4	0.724
22227	Ucp1	uncoupling protein 1 (mitochondrial, proton carrier)	2.507
225887	Ndufs8	NADH dehydrogenase (ubiquinone) Fe-S protein 8	0.504
230075	Ndufb6	NADH dehydrogenase (ubiquinone) 1 beta subcomplex, 6	0.501
235339	Dlat	dihydrolipoamide S-acetyltransferase (E2 component of pyruvate dehydrogenase complex)	0.314
269951	Idh2	isocitrate dehydrogenase 2 (NADP+), mitochondrial	0.862
407785	Ndufs6	NADH dehydrogenase (ubiquinone) Fe-S protein 6	0.329
56451	Suclg1	succinate-CoA ligase, GDP-forming, alpha subunit	0.774
66043	Atp5d	ATP synthase, H+ transporting, mitochondrial F1 complex, delta subunit	0.397
66046	Ndufb5	NADH dehydrogenase (ubiquinone) 1 beta subcomplex, 5	0.797
66142	Cox7b	cytochrome c oxidase subunit VIIb	1.024
66218	Ndufb9	NADH dehydrogenase (ubiquinone) 1 beta subcomplex, 9	0.545
66841	Etfdh	electron transferring flavoprotein, dehydrogenase	0.593
66925	Sdhd	succinate dehydrogenase complex, subunit D, integral membrane protein	0.567
67834	Idh3a	isocitrate dehydrogenase 3 (NAD+) alpha	0.644
68194	Ndufb4	NADH dehydrogenase (ubiquinone) 1 beta subcomplex 4	0.402
68197	Ndufc2	NADH dehydrogenase (ubiquinone) 1, subcomplex unknown, 2	0.326
68198	Ndufb2	NADH dehydrogenase (ubiquinone) 1 beta subcomplex, 2	0.461
68263	Pdhb	pyruvate dehydrogenase (lipoamide) beta	0.501
68342	Ndufb10	NADH dehydrogenase (ubiquinone) 1 beta subcomplex, 10	0.508
71679	Atp5h	ATP synthase, H+ transporting, mitochondrial F0 complex, subunit d	0.514
72900	Ndufv2	NADH dehydrogenase (ubiquinone) flavoprotein 2	0.365

76187	Adhfe1	alcohol dehydrogenase, iron containing, 1	-0.202
78920	Dlst	dihydrolipoamide S-succinyltransferase (E2 component of 2-oxo-glutarate complex)	0.437
